# Comparison of effects of HucMSCs, exosomes, and conditioned medium on NASH

**DOI:** 10.1038/s41598-023-45828-3

**Published:** 2023-10-27

**Authors:** Chenchen Liang, Siyuan Gao, Jianpeng Gao, Yanwen Xu, Qilong Li

**Affiliations:** 1 School of Public Health, Dali University, Dali, 671013 Yunnan China; 2 Center of Liver Diseases, The Third People’s Hospital of Kunming, Kunming, 650041 Yunnan China; 3 Department of Administration, Kunming Yan’an Hospital, Kunming, 650051 Yunnan China

**Keywords:** Stem cells, Zoology, Gastroenterology, Medical research, Molecular medicine, Pathogenesis

## Abstract

To investigate the effects and potential mechanisms of human umbilical cord mesenchymal stem cells, exosomes, and their conditioned media on lipid storage in oleic acid (OA) and palmitic acid (PA) treated hepatocytes and high-fat methionine- choline deficient diet (HFMRCD) induced non-alcoholic steatohepatitis (NASH) mice. AML12 cells were stimulated with OA and PA to establish the lipid storage cell model. HucMSCs, exosomes, and culture medium were then co-cultured. At the same time, C57BL/6 mice were fed an HFMRCD for 6 or 8 weeks to establish a NASH mouse model. The effect of HucMSCs, exosomes, and culture medium on lipid droplet repair of hepatocytes or NASH mice was then assessed. The weight of hepatocytes or liver tissue, Oil Red O, hematoxylin–eosin staining, Masson staining, Western blot, and qPCR were used to detect the related IL-6, TNF-α, TGF-β1 andEI24/AMPK/mTOR pathway expression in hepatocytes and liver tissue. Compared with the model group, the effect of HucMSCs-Ex on inhibiting the accumulation of lipid droplets was more obvious at the cell level. In vivo study showed that HucMSCs-Ex reduces activity scores in NASH mice and improves liver tissue morphology by reducing vacuolar degeneration, fat deposition, and collagen deposition of liver tissue. Western blot and qPCR results showed that inflammatory factors and AMPK/mTOR or EI24-related autophagy pathways were altered before and after treatment. HucMSCs, HucMSC-Ex, and CM can promote autophagy in hepatocytes or NASH mice through the AMPK/mTOR or EI24-related autophagy pathway and alleviate injury associated with lipid deposition, collagen deposition or inflammation, reversing the progression of NASH.

## Introduction

Non-alcoholic fatty liver disease (NAFLD) is a condition characterized by excessive lipid storage and degeneration of liver parenchymal cells that range from more benign condition of non-alcoholic fatty liver (NAFL) to severe non-alcoholic steatohepatitis (NASH). Excessive development of NASH can lead to liver fibrosis or hepatocellular carcinoma; therefore, timely interruption or reversal of NASH is very important to patients and public health^1–3^. According to recent meta-analysis studies (1990–2019), the overall global prevalence of NAFLD reached 30.05^4,5^. The highest overall prevalence of NAFLD is found in Latin America (44.37%), followed by the Middle East and North Africa (MENA) (36.53%), South Asia (33.83%), and South East Asia (33.07%)^6–8^. Also, eighteen studies have reported an overall pooled prevalence of NAFLD of 50.9/1000 person-years (95% CI: 44.8–57.4) in mainland China [n = 8], Korea [n = 8], Hong Kong [n = 1] and Japan [n = 1], with mainland China having the highest prevalence of NAFLD (63.0/1000 person-years [47.0–81.3]) and Japan having the lowest prevalence (29.0/1000 person-years [26.3–31.7])^9^.

The complexity of its pathogenesis and the uncertainty of disease assessment have been hindering pharmaceutical research and development in NASH^10^. In the past decade, umbilical cord mesenchymal stromal cells (HucMSCs), their exosomes (HucMSC-Ex), and conditioned medium (HucMSC-CM) have been widely used as regenerative medicine to treat various medical conditions. Several preclinical and clinical studies have shown that mesenchymal stromal cells (MSCs) transplanted from bone marrow, umbilical cord, and adipose tissue have therapeutic potential in steatohepatitis, chronic liver fibrosis and other liver disease^11–14^. A recent animal study showed that MSCs might be used to treat NASH induced by an HFMRCD, which was proven by decreased high-fat diet-induced weight gain, expansion of subcutaneous adipose tissue, steatosis, lobular inflammation and liver fibrogenesis^14^. Yet, MSC-based therapies have also been linked with certain side effects, including potential tumourigenic, teratogenicity, and infection potential^15^.

Paracrine signals are believed to mediate the immunomodulatory function of MSCs^16^. Among them, HucMSCs-Ex, an important manifestation of paracrine signaling in HucMSCs, is considered a promising approach for cell-free therapy as it exerts multiple biological activities and intercellular communication functions^17^. HucMSCs work as a natural drug delivery vehicle that can target specific tissues and are associated with fewer adverse effects^18^. Moreover, the conditioned medium solution for HucMSCs is a cell-free and serum-free supernatant containing high levels of growth factors and cytokines that have been widely used in immune disorders, fatty liver, osteoarthritis, chronic trauma, and alopecia treatment^18–21^. MSC-CM can improve insulin resistance in NAFLD mice, amend the pathological structure of the liver, enhance the liver's total antioxidant capacity and mitochondrial function, and reduce inflammation and cell apoptosis^22^. These findings provide novel indications that MSC, MSC-Ex, and MSC-CM can potentially treat patients with NAFLD clinically.

**Figure 1:**
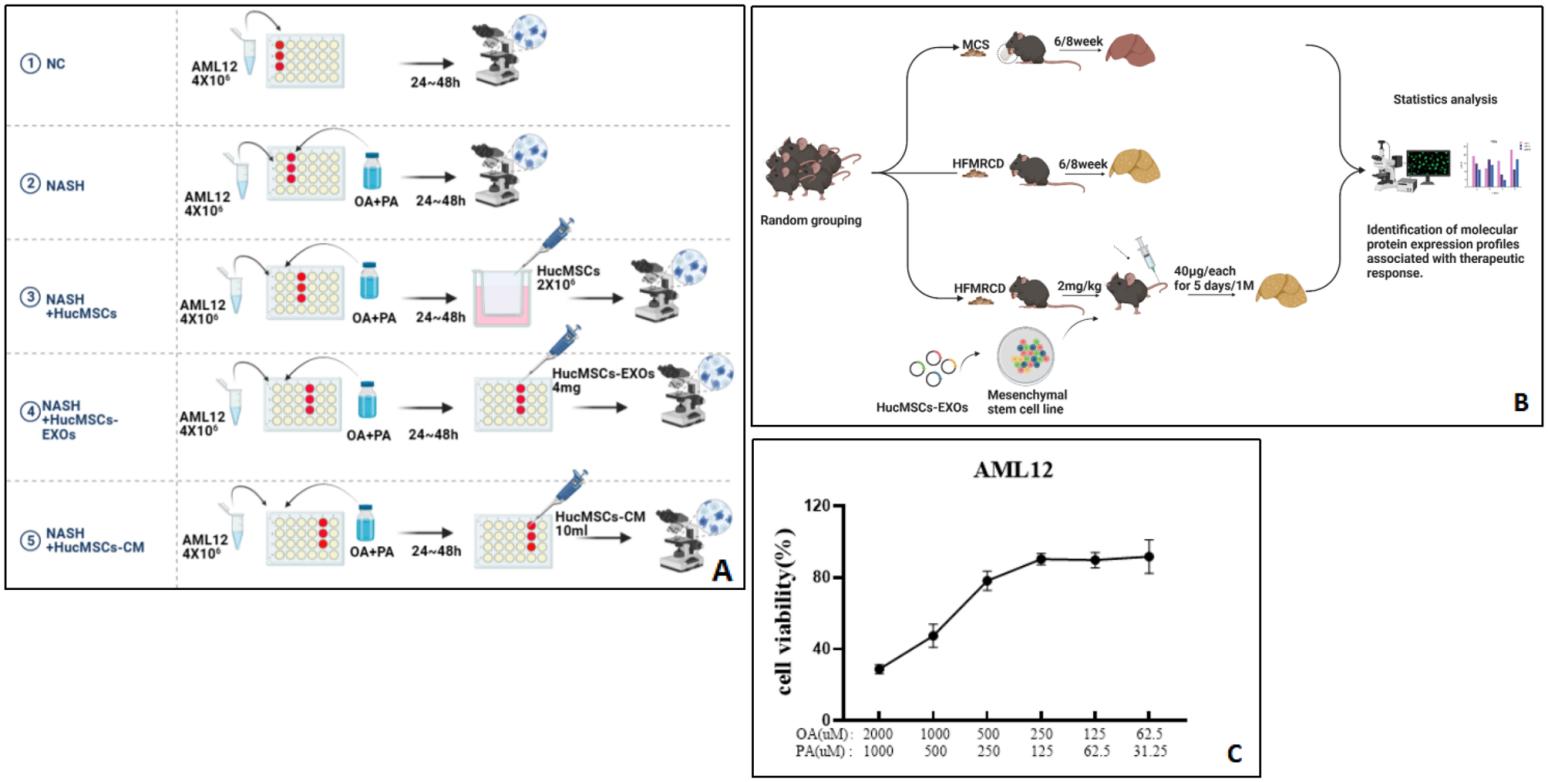
(**A**) Cell grouping and experimental interventions; (**B**) Construction of a mouse NASH model and therapeutic intervention; (**C) **Trends in cell viability.

This study further verified the therapeutic effects of HucMSCs, exosomes, and conditioned media by establishing NASH mouse animal models and hepatocyte lipid storage models. Firstly, a hepatic parenchymal cell lipid storage model was established in vitro using a mixture of OA and PA drugs stimulated mouse hepatocyte lines (*AML-12*). Subsequently, HucMSCs, HucMSCs-Ex, and conditioned media were co-cultured with the hepatocyte lipid storage model using the 2D model to verify whether HucMSCs, HucMSCs-Ex, and conditioned media could effectively improve the physiological, biochemical, and pathological characteristics of NASH, and to explore their efficacy on NASH at the cellular level (Fig. 1). Afterward, in vivo model of NASH was established by HFMRCD feeding. HucMSCs-Ex was transplanted into the NASH animal model by tail vein injection (Fig. 1). In vivo experiment was used to clarify whether HucMSCs-Ex could effectively improve the relevant indexes of NASH. Meanwhile, we also found that the AMPK-activated protein kinase (AMPK/mTOR) signaling pathway had a crucial role in the regulation of lipid metabolism mediated by HUC-MSCs or exosomes^23^, which could attenuate glycolipid metabolism dysfunction by activating AMPK expression^24^, thus contributing to ameliorating or reversing lipolysis or inflammation levels in OA/PA-treated hepatocytes or liver of NASH mice. The above provided theoretical support for treating NASH or other Inflammatory immunometabolic diseases with HucMSCs, HucMSCs-Ex, and conditioned medium solution.

## Results

### Characterization of HucMSCs and HucMSCs exosomes

**Figure 2:**
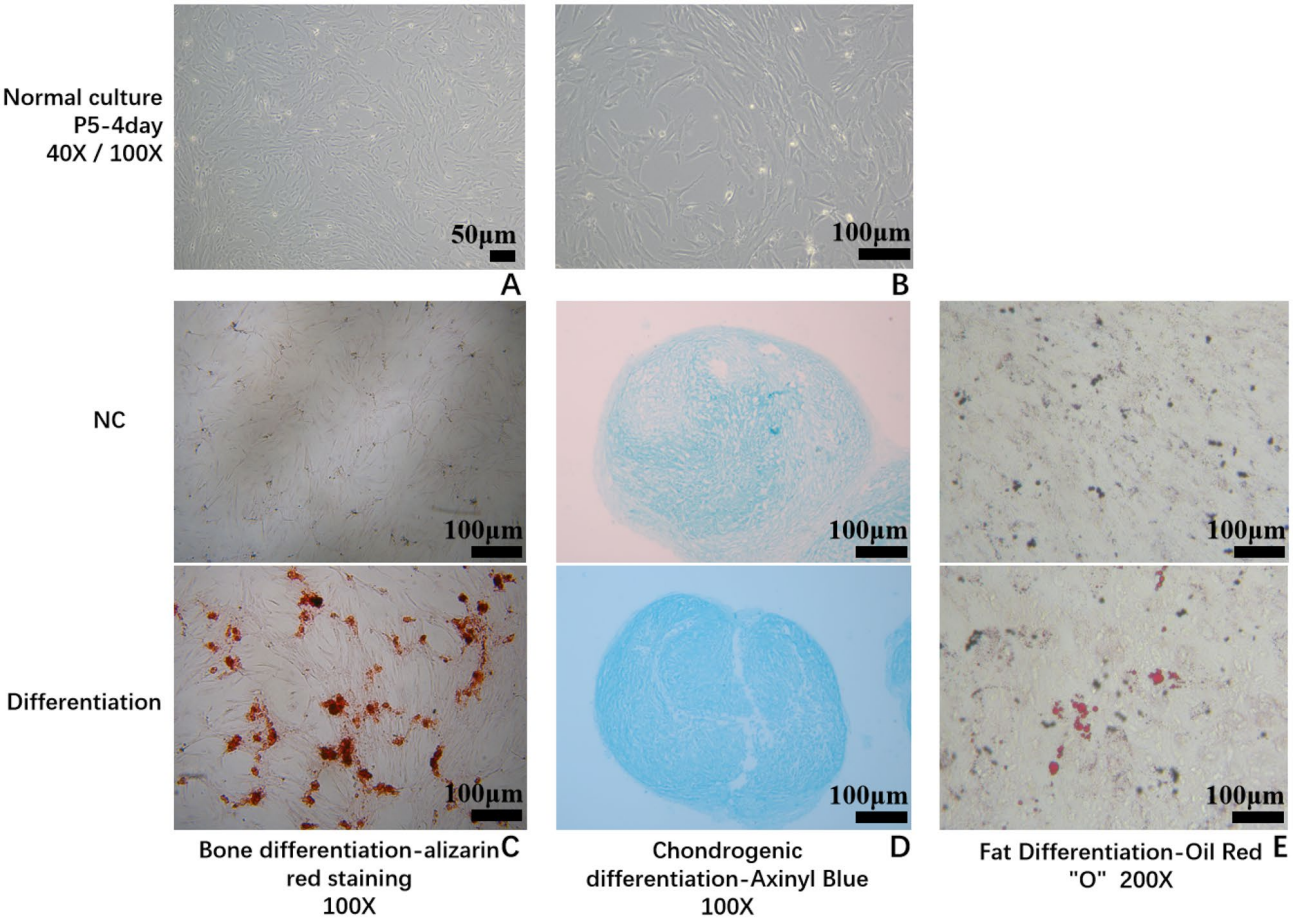
Characterization of hUC-MSCs was performed. (**A**) Normal culture-P5-4 day, 40X; (**B**) Normal culture-P5-4 day, 100X; (**C**) osteogenesis of Huc-MSCs, 100X; (**D**) chondrogenic differentiation of Huc-MSCs; (**E**) lipogenic differentiation of Huc-MSCs.

**Figure 3:**
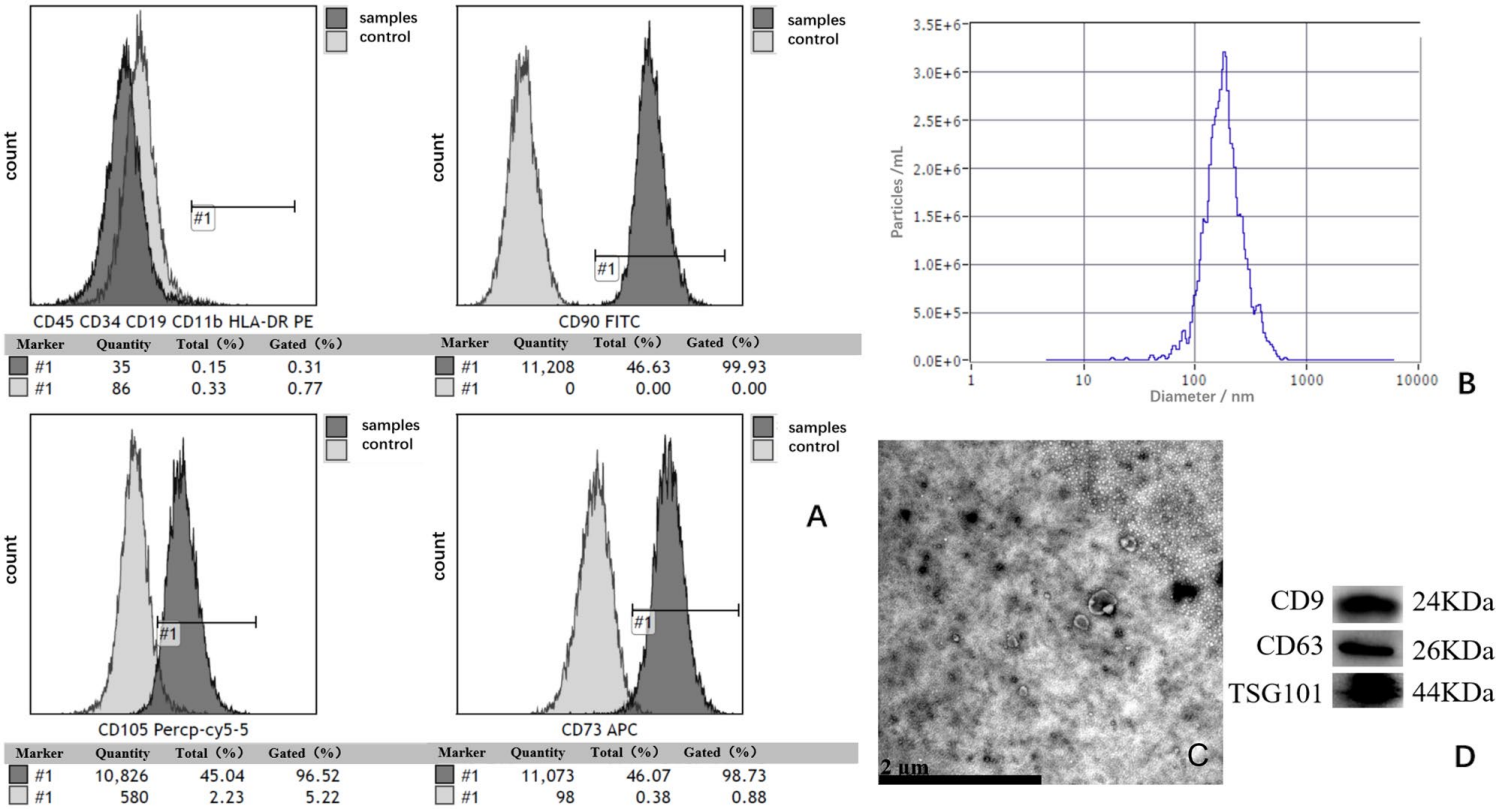
Flow characterization of MSC surface markers and Identification of HucMSC-derived exosomes. (**A**) The surface markers of hUC-MSCs were examined by flow cytometry (CD90, CD105, CD73, CD45, CD34, CD19, CD11b and HLA-DR); (**B**) Particle size distribution of Huc-MSCs exosomes determined by NTA (1.4E+11 Particles/mL); (**C**) TEM image showing the morphology of Huc-MSCs exosomes, 20 K; (**D**) Expression of Huc-MSCs exosomes CD9, CD63 and TSG101 determined by Western blotting. (Note: CD9, CD63 and TSG101 groups using multiple exposures; and blots or gels have been cropped, the CD9, CD63 and TSG101 original blots or gels are shown in the Supplementary Fig. 27).

HucMSCs were identified by flow cytometry analysis according to the criteria of the "Mesenchymal and Tissue Stem Cell Committee of the International Society for Cellular Therapy"^16^ to obtain HucMSCs or exosomes. As shown in Fig. 3A, most HucMSCs expressed high levels of CD90, CD105, and CD73, whereas the percentage of CD45, CD34, CD19, CD11b and HLA-DR-positive cells was very low. Figure 2 shows the morphology of primary cells in normal culture and the ability of these HucMSCs to differentiate into osteoblasts, adipocytes, and chondrocytes.

After the collection and isolation (Fig. 3C), exosomes presented as small disc-shaped membranous vesicles with bilateral membrane structures under a transmission electron microscope (TEM). The particle size distribution of the exosomes was determined by nanoparticle Tracking Analysis (NTA) (Fig. 3B), and and analysis showed that the 1.4E+11 particles/mL exosomes were approximately 110 nm in diameter. Western blot detected markers characteristic of exosomes from HucMSCs, including CD9, CD63, and TSG101 (Fig. 3D). In conclusion, HucMSCs exosomes that met the criteria were successfully isolated from culture supernatants^25,26^.

### HucMSCs, HucMSCs-Ex, and HucMSCs-CM, reduce OA PA-induced lipid storage in hepatocytes

**Figure 4:**
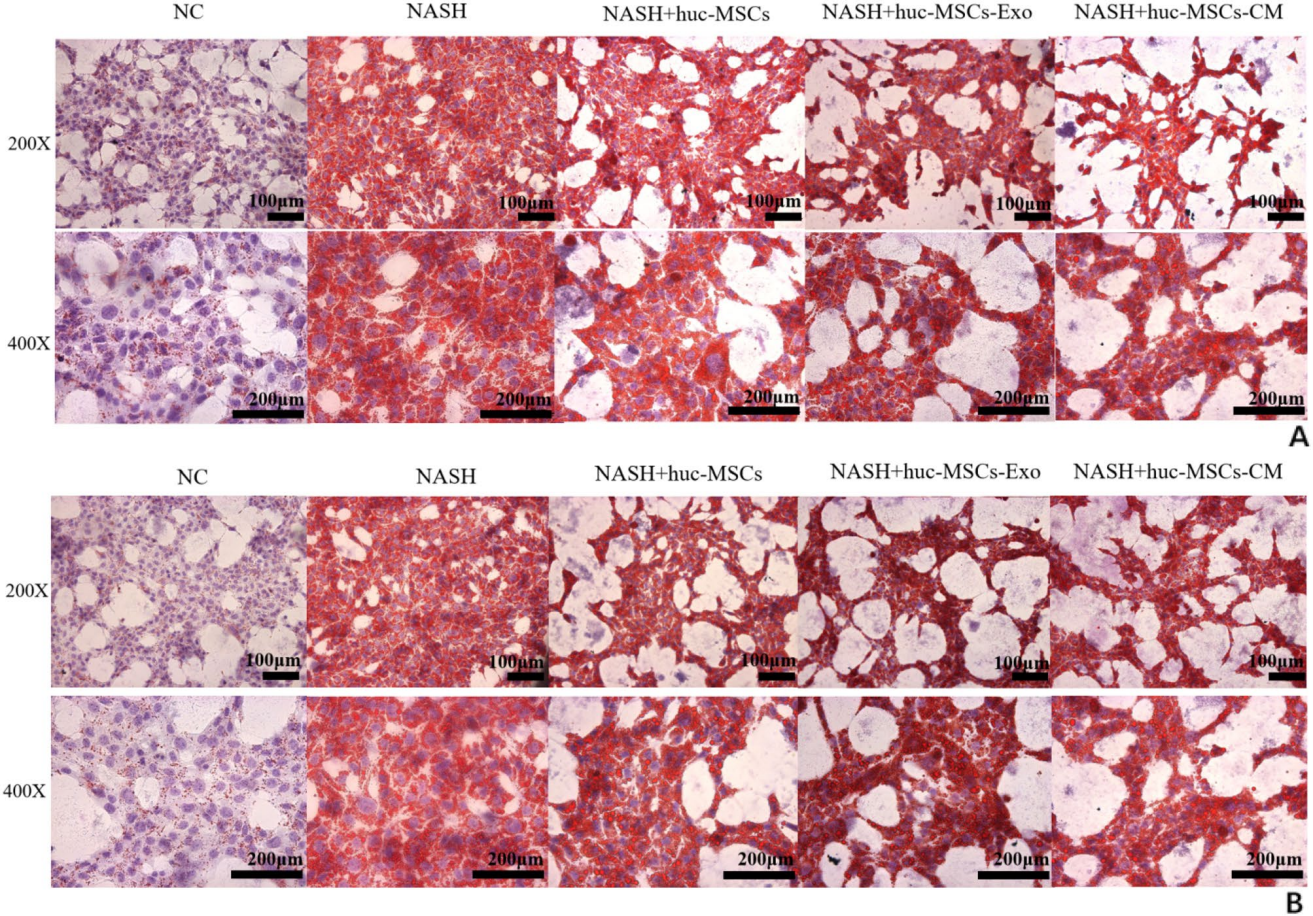
(**A**) Levels of lipid storage in the cell model after 24 h co-culture; (**B**) Levels of lipid storage in the cell model after 48 h co-culture.

The NASH hepatocyte lipid storage model was established by the OA and PA methods. HucMSCs were cultured in the Transwell upper chamber, and the in vitro cell model was cultured in the lower chamber. After co-culturing for 24–48 h, the co-cultured lower chamber cells were stained with Oil Red O to detect the level of lipid storage in the cell model after co-culture, followed by a collection of cellular molecules or proteins to detect the expression level of relevant inflammatory factors. The reduction of lipid droplets was not obvious in 24 or 48 h HucMSCs-treated groups; yet, the number of droplets was reduced in HucMSCs-Ex or CM groups compared to the HucMSCs group, even though the observed reduction was not significant (Fig. 4, *P* < 0.05).

**Figure 5:**
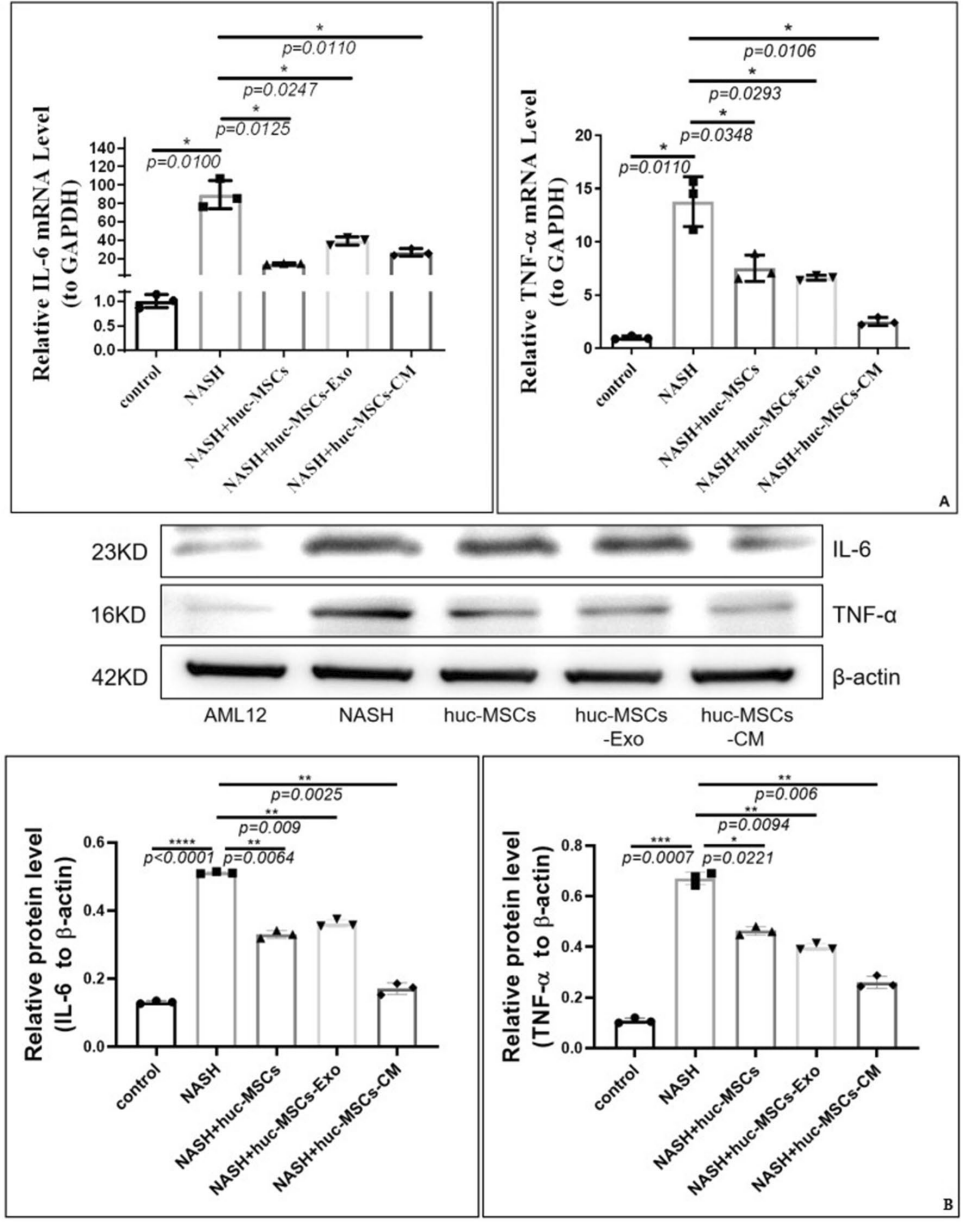
(**A**) Changes in mRNA expression levels of related inflammatory factors such as IL-6 and TNF-α; (**B**) Changes in protein expression levels of related inflammatory factors such as IL-6 and TNF-α (Note: This gel blot was cut in half for subsequent protein detection prior to antibody hybridization; and blots or gels have been cropped, the IL-6, TNF-α and β-actin original blots or gels are shown in the Supplementary Fig. 28; IL-6, TNF-α and β-actin groups using multiple exposures; the multiple exposure images can be seen in Supplementary Fig. 37).

Next, we performed the subsequent statistical analysis of the quantification of lipid droplets, and the results showed that the exosome and CM groups could inhibit the accumulation of lipid droplets more effectively (Figure S2 online, P < 0.05). Moreover, we also found that the expression levels of IL-6 and TNF-α were decreased in HucMSCs, HucMSCs-Ex, and CM co-culture cell models (Fig. 5, all *P* < 0.05). Therefore, we concluded that the inhibitory effect of the HucMSCs-Ex and CM groups is superior to the HucMSCs group.

### Analysis of mice body weight and liver wet weight

**Figure 6:**
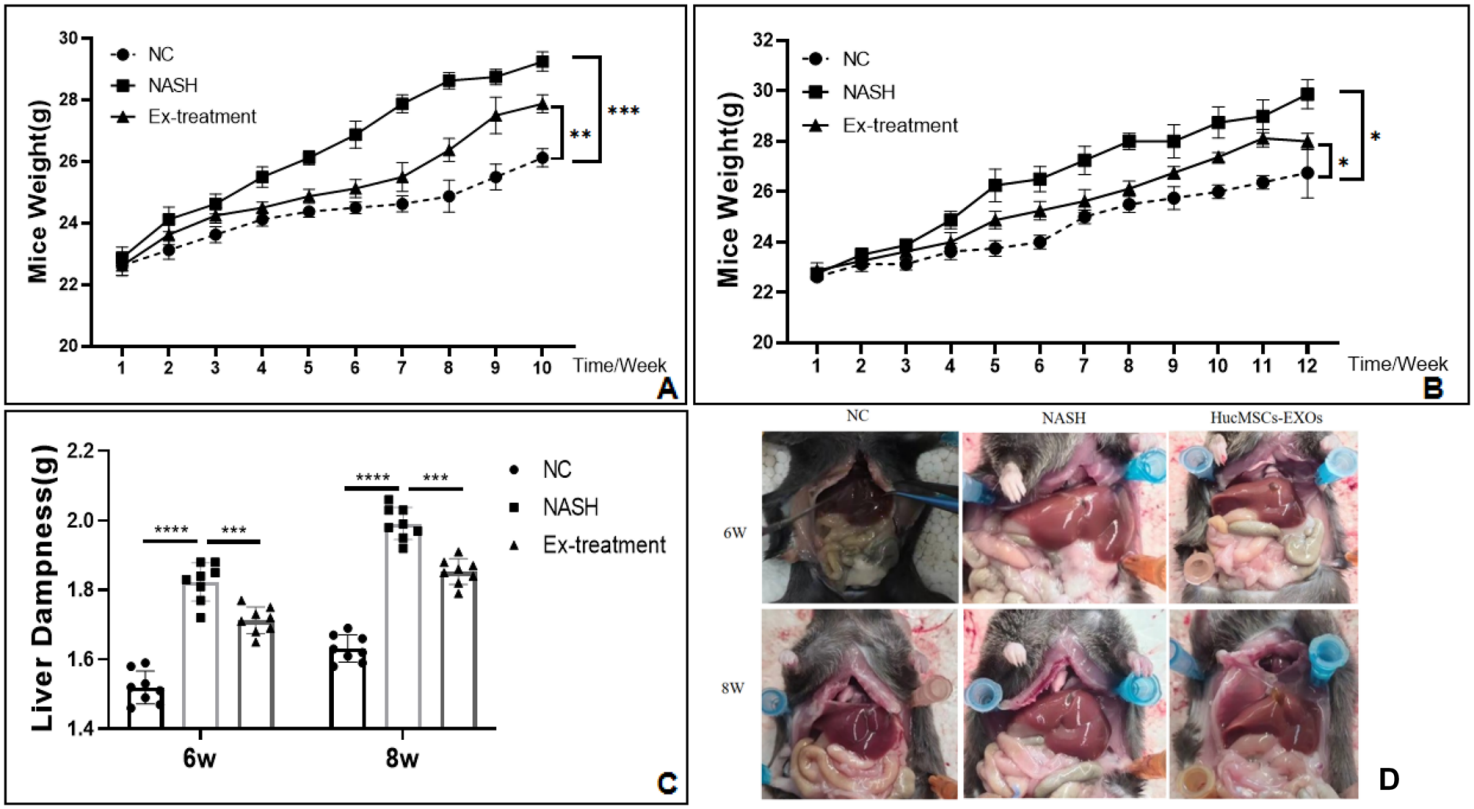
(**A**) Analysis of body weight changes of mice in each group at week 6 (**p < 0.01); (**B**) Analysis of body weight changes of mice in each group at week 8 (*p < 0.05); (**C**) Analysis of Liver Dampness (***p < 0.001); (**D**) Morphological analysis of mouse liver.

No mice died during modeling and treatment, and 48 mice were included in the final analysis. As shown in Fig. 6, the body weight of mice in each group increased at weeks 6 and 8; yet, the body weight of the NASH mice increased on week 2 at a higher rate compared to the control and treated mice (Fig. 6A, B, all *P* < 0.05). At the same time, the wet liver weight of mice in the normal control group continued to increase, while the wet liver weight of mice in the model group continued to increase at a higher rate than that of the control and treated groups (Fig. 6C, all *P* < 0.05).

### HucMSCs-Ex improves liver tissue morphology and reduces activity scores in NASH mice

**Figure 7:**
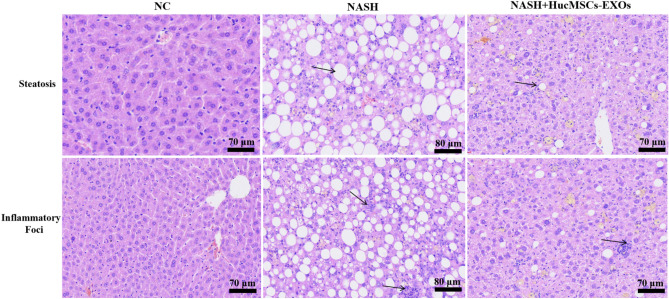
6thW HE staining (fat vacuoles, inflammatory lesion, 400X).

**Figure 8:**
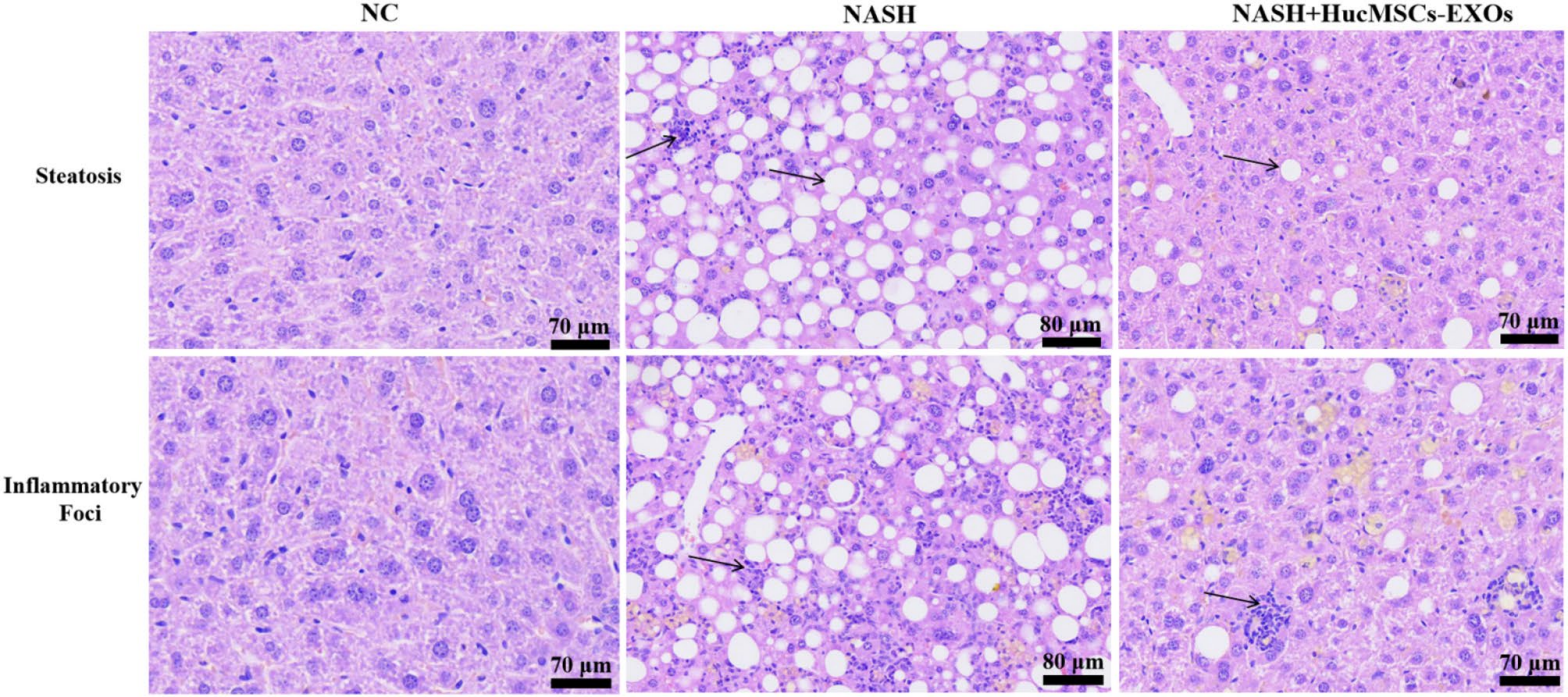
8thW HE staining (fat vacuoles, inflammatory lesion, 400X).

Next, we examined the effect of HucMSCs-Ex treatment on NASH mice after 1 month of treatment by gross morphological observation of the liver and pathological HE staining in each group, and the results showed that the livers in the Ex-treated group were slightly smaller and darker compared with the NASH group, but still with a fatty liver (Fig. 6D). Also, the number of fatty vacuoles and the amount of inflammatory necrotic foci were significantly reduced in the liver tissue of HucMSCs-Ex treated mice compared to the NASH group (Figs. 7, 8 and Supplementary Fig. 3 online).

NASH is often associated with collagen or extracellular matrix deposition, which promotes NASH fibrosis or cirrhosis^27^. In this study, Masson's staining showed that the collagen deposition was significantly reduced, and the staining was lighter in the liver tissue in the HucMSCs-Ex treatment group compared with the model group (Supplementary Figs. 1, 4 online). In addition, QP or WB assays for molecular or proteomic transfection efficiency showed significantly decreased IL-6, TNF-α, TGF-β1 mRNA, and protein levels in HucMSCs-Ex group mice were significantly decreased (Supplementary Figs. 5–9 online).

In addition, the "NAS" activity score of the model mice was > 5 (*P* < 0.05); "NAS" activity score in the HucMSCs-Ex group was reduced to approximately 2 points compared to the model group (Supplementary Table 2, *P* < 0.05).

All these results indicated that NASH mice were successfully modeled at weeks 6 and 8 and that HucMSCs-Ex improved liver tissue structure and reduced "NAS" activity scores in NASH mice.

### AMPK/mTOR signaling pathway has an important role in the inhibition of NASH by HucMSCs, HucMSC-Ex, and CM

To explore the potential mechanism of the possible effect of HucMSCs, HucMSC-Ex, and CM, we investigated three possible AMPK/mTOR/EI24 pathways. The obtained results showed that at the cellular level, the AMPK mRNA and protein levels were effectively increased in the HucMSCs, HucMSC-Ex, and CM groups, while mTOR mRNA and protein levels were significantly inhibited in these groups; the most pronounced effect was seen in the exosome group (Supplementary Figs. 10–12 online). Similarly, in vivo study suggested that mTOR mRNA and protein levels were inhibited, while AMPK levels were significantly increased (Supplementary Figs. 10–17 online).

Next, we investigated whether HucMSC-Ex could regulate the AMPK/mTOR pathway. Western blot results showed that hucMSC- Ex increased p-AMPK protein levels and inhibited p-mTOR levels compared to the NASH group (Supplementary Fig. 13 online). AMPK, as an important kinase in the biological metabolism of organisms and eukaryotic cells, is mainly involved in accelerated fatty acid oxidation (FAO), autophagy, and degradation of cholesterol and lipid^28,29^. Therefore, targeting the regulation of AMPK may be beneficial for treating NASH.

EI24 (etoposide-inducible 2.4 protein) is closely associated with the activation of the adenosine monophosphate-activated kinase (AMPK)/mammalian target of rapamycin (mTOR) axis and can promote the degradation of autophagic lysosomes, thus exerting a protective effect on autophagy^30^. Accordingly, we examined the upstream of AMPK regulation and EI24 levels in liver tissue or hepatocytes. The results showed that EI24 was significantly upregulated in the NASH group, and EI24 levels were significantly overexpressed after treatment (Supplementary Figs. 20–25 online). Similarly, in vivo study showed that p-EI24 protein levels were significantly increased after HucMSC-EXO action. Thus, we hypothesized that HucMSC-Ex may also exert a therapeutic effect related to NASH by upregulating EI24 (Supplementary Fig. 26 online).

Finally, we detected membrane marker proteins associated with downstream regulation of the AMPK/mTOR pathway in autophagosomes. The results showed that HucMSCs, HucMSC-Ex, and CM had increased LC3BII/I ratio and significantly down-regulated P62 protein levels (Supplementary Figs. 10–12 online), while the HucMSCs-Ex group in vivo showed a similar trend, indicating increased LC3BII/I ratio and significantly decreased P62 protein levels (Supplementary Figs. 16–19 online).

## Discussion

In recent years, NAFLD has become an important risk factor for many metabolic diseases, often preceding more serious conditions such as NASH, cardiovascular disease, type 2 diabetes (T2D), cirrhosis, and hepatocellular carcinoma (HCC)^31,32^. Despite extensive research, there are currently no effective therapies for NAFLD^33^. With the emergence of various therapeutic trial studies using HucMSCs, exosomes, and CM, the role of HucMSCs and exosome-based transplantation in treating lipid metabolism in metabolic diseases has become a hot topic. A systematic review reported that using umbilical cord MSCs and exosomes as a medium for treating obesity-related metabolic diseases may be a promising approach^34^. However, there are inherent risks associated with the use of HucMSCs.

Exosomes, an important part of paracrine signaling in HucMSCs, are considered a more effective cell-free therapy because they have multiple biological activities and intercellular communication functions and are easier to quantify and maintain biological activities during storage and transport. Previous studies have shown that exosomes derived from HucMSCs have similar therapeutic effects to HucMSCs and can avoid the risks associated with HucMSC injections^35^. However, there is very little evidence of the above interventions for NASH using conditioned media solutions of HucMSCs in addition to the use of adult stem cells or exosome transplantation^18^. In our study, three treatment groups of HucMSCs, exosomes, and CM were established at the cellular level to find the best treatment protocol at the animal level. The final results showed that all three treatment groups inhibited OA and PA-induced lipid storage in hepatocytes, but the inhibition effect was more pronounced in the HucMSCs-EXOs group than in the HucMSCs and HucMSCs-CM groups (Fig. 4 and Supplementary Fig. 2 online). Similar results were reported by Hui et al. who found that ADSC-EVs treatment ameliorated obesity and reduced liver steatosis in an obese mouse model^36^.

Subsequently, our results showed a prominent decrease in liver "NAS" score index in mice after one month of maintenance treatment with six 40 μg HucMSCs-Ex tail vein injections (*P* < 0.05, Supplementary Table 2). In addition, HucMSCs-Ex treatment reduced body weight, liver wet weight, lipid deposition, and collagen deposition in NASH mice (Figs. 6–8 and S1–S4). Remarkably, the body weight of the treated group was lower than that of the "NASH" group before treatment (by week 7 or 9). We hypothesized that there may be some limitations in this section, which may be related to individual differences in the mice. However, we then carefully analyzed the difference in body weight growth rate before and after treatment in weeks 7–10 and 9–12, and found that the weight growth rate of the treatment group slowed down in week 8, but the value of the difference in growth rate in week 6 was not satisfactory (Supplementary Tables 4–5). We hypothesized that there would probably be a trend of decreasing body weight growth rate only from week 8. Meanwhile, We also analyzed the wet weight data of the mouse liver and found that the growth rate was slowed in the treatment group. (Supplementary Table 6). In a previous study, Hou et al*.* found that NASH was associated with the elevation of many cytokines, especially IL-6 and TNF-α^37^. Similarly, in this study, the levels of IL-6 and TNF-α were increased by the HFMRCD-induced NASH mouse model as well as by OA and PA-treated hepatocytes, while IL-6, TNF-α or TGF-β1 levels were downregulated (Fig. 5 and Supplementary Figs. 5–9). Thus, whether HucMSCs-Ex carries certain factors that can inhibit IL-6 and TNF-α levels in NASH mice and alleviate NASH needs to be further investigated.

Although the role of HucMSCs-Ex in NASH is still unclear, considering that HucMSCs-Ex contains a variety of metabolism-related enzymes, we observed the therapeutic role of HucMSCs- Ex in NASH through HFMRCD-induced NASH Mice model and OA- and PA-treated hepatocytes. First, we analyzed the relevant signaling pathways in vivo, finding significant enrichment of AMPK-related signaling pathway factors at mRNA and protein levels after HucMSCs-Ex action (Supplementary Figs. 14–17 online, *P* < 0.05). In HucMSC-Ex treatment group at 6W or 8W, exosome increased p-AMPK protein levels and inhibited p-mTOR levels compared with the NASH group (Supplementary Fig. 13 online, *p* < 0.05).

AMPK/mTOR (mammalian target of rapamycin) is the central molecule controlling cellular autophagy that regulates autophagy initiation by controlling the activity of the autophagy initiation ATG13/ULK1 (Unc-51-like kinase) kinase complex [including ULK1, FIP200 (FAK family interacting protein of 200 kDa), ATG13 and ATG101] to regulate autophagy^38^. It has been shown that mTOR overactivity is closely associated with fatty acid synthesis, hepatic insulin resistance, and type 2 diabetes^39^. Activation of mTOR can phosphorylate Unc-51-like autophagy-activated kinase 1 (ULK1) and autophagy-associated protein 13, thereby inhibiting autophagy^40^. In contrast, the AMPK pathway participates in lipophagy or degradation in vivo by regulating the process of cellular autophagy initiator formation, somatic membrane extension, and maturation by activating kinase activity in response to ULK1 complex dephosphorylation to accelerate autophagy. Meanwhile, LC3 protein, a recognized marker of autophagy initiation, and P62, autophagy-specific substrates, both can be determined by Western blot.

In this study, we established three treatment groups of HucMSCs, exosomes, and medium solution at the cellular level. mTOR mRNA and protein levels were inhibited, while AMPK levels were significantly increased, LC3BII/I ratio was increased, and P62 levels were significantly decreased in all three groups compared to the control group (Supplementary Figs. 10–12 online, *p* < 0.05). Similar results were obtained at the animal level, where HucMSCs-Ex significantly inhibited mTOR and P62 mRNA expression, decreased mTOR and P62 protein levels, and increased AMPK and LC3BII/α protein levels in mice treated with HucMSCs-Ex (Supplementary Figs. 16–19 online, *p* < 0.05). Meanwhile, liver tissue steatosis, inflammation, and other collagen deposition liver damage significantly reduced in mice after HucMSCs-Ex intervention (Figs. 7–8 and Supplementary Figs. 1–4 online).

We also examined the autophagic transmembrane protein EI24 expression levels in liver tissue or hepatocytes. The results at the cellular level showed that EI24 was significantly elevated in NASH, and the level of EI24 was significantly overexpressed after treatment (Supplementary Fig. 20 online); meanwhile, at the animal level, the p-EI24 protein level was significantly increased after the action of HucMSC-Ex (Supplementary Figs. 21–26 online). It has also been speculated that HucMSC-Ex may exert a therapeutic effect related to NASH by upregulating EI24.

The above results suggest that HucMSCs-Ex may act on more than one pathway and may promote lipid degradation, reduce lipid synthesis, or activate autophagic flux level to accelerate lipid degradation or fatty acid synthesis by regulating AMPK/mTOR signaling pathway or upregulating EI24 protein levels to achieve NASH prognosis improvement.

While experimental studies on stem cells and their exosomes are flourishing, they have not yet been widely used clinically, mainly due to the lack of practical clinical applications. In addition, the active molecules functioning in exosomes and their mechanism of action have not been fully understood. Subsequent studies on high-throughput sequencing, both in vivo and in vitro*,* are still needed to discover more relevant gene expression changes, explore the mechanism of action of HucMSCs-Ex in inhibiting NASH liver disease, and provide new therapeutic strategies for the clinical treatment of NASH.

## Materials and methods

### Materials

Fetal Bovine Serum (Bio-Channel, BC-SE-FBS07), DMEM/F-12 (BiologicalIndustries, 01-170-1A), EDTA-Trypsin (Biosharp, BL512A), EndoFectin™Max Reagent (GeneCopoeia, EF013), Cell Value Added-Toxicity Assay Kit (Biosharp, BS350B), PA (Aladdin, S161420), OA (Maclean, S817542), ORO Staining Kit (Solarbio, G1262), BlazeTaq™ SYBR®Green Mix (GeneCopoeia, QP031), Dnase QP031), BCA Protein Reagent (P0009-1, P0009-1), Hematoxylin (Solarbio, H8070), Eosin Staining Reagent (Beyotime, C0105-2), Masson Trichrome Staining Reagent (Solarbio, G1340), TRIzol®Reagent (Life, Cat. no. 15596-018), SureScript™ First-Strand cDNA Synthesis Kit (GeneCopoeia, QP056), Immobilon Western HRP Substrate Luminol Reagent (Affinity, KF001), 30% Acrylamide/Bis solution (29:1) (Solarbio, A1010), 24-well plate (VIRYA, 3512409), FBS(Bio-Channel, BC-SE-FBS07), Hieff® Quick exosome isolation kit Plus (YEASON, China).

Tissue embedding kit (Jiangsu Shitai, 20084), Real-timePCR (Thermo Fisher Scientific, TCR0096, USA), UV spectrophotometer (ALLSHENG, USA), benchtop low-speed centrifuge (Weil Ltd., China), inverted fluorescence microscope (Zeiss, Observer.A1, Germany), gel imager (Monad, USA), ice maker (FM150KE, China), 4 °C refrigerator (Haier, China), baking machine (Thermo Scientific, DB-B2, USA), TEM(ZCIBIO, China), NTA(Particle Metrix, China).

### Experimental cells

In this experiment, 5-week male AML-12 mouse hepatocyte lines were selected from Power Science Biotechnology Co. and stored and stably cultured for long periods at Quanport Biological Laboratory. The cells were cultured in DMEM/F12 complete medium (BiologicalIndustries, 01-170-1A), passaged on alternate days, and incubated in a constant temperature incubator at 37 °C and 5% CO_2_.

### Lab animals

#### Mice

48 specific pathogen Free (SPF) male 6–8 week old C57BL6 mice, weighing 20–25 g, 8 per cage, were purchased from Hunan Slaughter Jingda Laboratory Animal Co. The mice were housed in the experimental animal house of the Institute of Medical Biology, Chinese Academy of Medical Sciences [SYXK (Yunnan) K2022-0006] during the experimental period; the housing environment: temperature 22 ± 1 °C, humidity 65 ± 5% with half-cycle lighting at day and night; the mice were acclimatized and fed for 1 week before the experiment; (All authors confirmed the study is reported in accordance with ARRIVE guidelines;All experiments and methods were carried out in accordance with relevant guidelines and regulations;All procedures performed in studies involving animals were in accordance with the ethical standards of the institution at which the studies were conducted and ethical approval was obtained from [Experimental Animal Ethics Committee of Kunming Yan'an Hospital, 2023004]).

#### Feed

SPF grade mouse high-fat methionine-choline deficient model feed (HFMRCD) (AIN-76) provided by Beijing Co-operative Feeds Ltd [SCXK (Beijing) 2019-0003] and bedding provided by Jiangsu Medison Biomedical Co; (The relevant human umbilical cord-derived mesenchymal stem cells, exosomes and conditioned culture mediums required for this study were kindly provided by the Central Laboratory of Yan'an Hospital, Kunming, Yunnan Province.)

### Cultivation and characterization of hUC-MSCs and CM

The hUC-MSCs were maintained at the Central Laboratory of Yan'an Hospital affiliated with Kunming Medical University, placed in DMEM/F12 complete culture flasks with 10% FBS(Bio-Channel, BC-SE-FBS07), and cultured in a constant temperature incubator at 37 °C and 5% CO2. After the cells were attached to the wall, their adhesion was observed, digested and counted, and they were inoculated into 24-well (VIRYA, 3512409) culture plates at a density of 4X10^4^cells/well. After 2–3 days of culture, cell growth was observed separately. After replacing the lipid-induced medium, osteogenic-induced medium, or chondrogenic-induced medium in a CO_2_ incubator at constant temperature, the medium was changed every 2–3 days. On the 16th or 21st day of induction culture, the cells were stained with oil red O or alizarin red or toluidine blue for 5 min, washed twice with pure water, and observed and photographed under an inverted microscope (Zeiss, Observer.A1, Germany). Finally, the CM was collected and stored at 4 °C refrigerator (Haier, China), and the collected hUC-MSCs were subjected to flow cytometry to detect the surface markers CD90, CD105, CD73, CD45, CD34, CD19, CD11b and HLA-DR.

### Isolation and characterization of hUC-MSCs exosomes

The hUC-MSCs were provided by the Central Laboratory of Yan'an Hospital, Kunming Medical University. hUC-MSCs were cultured in Dulbecco's modified Eagle's medium (DMEM; Gibco, USA), and the adherent cells were incubated in 10% fetal bovine serum (FBS)(Bio-Channel, BC-SE-FBS07) under cell incubation conditions (37 °C and 5% CO2) for at least 24 h. Then, the hUC-MSCs culture supernatant was collected, centrifuged(Eppendorf, Centrifuger5418), filtered and concentrated to remove dead cells, cell debris and decontamination. The hUC-MSCs exosomes were extracted from the cell supernatants using the Hieff® Quick exosome isolation kit Plus (YEASON, China) according to the kit instructions, resuspended in sterile PBS(Solarbio, NO. P1003), and stored at − 80 °C (Haier, DW-86L626). The protein concentration of exosomes was determined using the BCA protein assay kit (beyotime, P0009-1). The extracted hUC-MSCs exosomes were characterized by transmission electron microscopy (TEN; ZCIBIO, ZC1099). Particle size was analyzed by nanoparticle tracking analysis (NTA; Particle Metrix, ZetaView), and Western blot was used to detect exosome surface markers TSG101, CD9 and CD63.

### In vitro model construction of NASH lipid storage

First, AML-12 cells were induced with 2 mM oleic acid (OA) (Maclean, S817542) and 1 mM palmitic acid (PA) (Aladdin, S161420), in sequential fold dilutions for 48 h. Then add 100 μl of medium to 96-well plate (Thermo Fisher Scientific, ASF-0020B5-F10) (30 wells), followed by 50 μl of configured OA preliminary dilution + 50 μl of PA preliminary dilution to B5-F5 (5 wells), sequential dilution (see Supplementary Table 1). The medium in the plate was then aspirated and discarded, and 100 μl of cell medium was added to B2-F3 (10 wells) as a blank control group and a cell control group according to grouping; 100 μl of diluted NAOH medium was added to B4-F4 (5 wells) as a solvent control group; AML12 cells in good growth condition and in logarithmic growth phase were digested and counted, and 1.5 × 10^5^ cells were taken Suspended into 5 ml (30,000 cells/1 ml) and mixed, 100 μl of cell suspension (3000 cells) was added to B3-F10 (in 40 wells) in the plate. Finally, the drug-treated 96-well plate was placed in the incubator and incubation continued for 48 h (note the cell gradient). 10 μl cck8 (Biosharp, BS3508) was added to each well and measurement of absorbance at 450 nm using an enzyme meter (Biotek, ELX800) after 2 h of incubation (see Fig. 1C and Supplementary Table 1).

### Cell grouping and experimental intervention

AML-12 cells in logarithmic growth phase were digested and counted and randomly divided into A: Normal, B: NASH model, C: NASH + HucMSCs, D: NASH + HucMSCs-Ex and E: NASH + HucMSCs-CM treatment groups and spread in 24-well plates (VIRYA, 3512409) according to 4X106 AML-12 cells, Groups A, B, C, E 3 wells per group, and one T-25 vial for group D. 1.after the cells were attached to the wall, the medium of groups B, C, D and E was replaced with drug-added medium oleic acid (OA: 500uM)) and palmitic acid (PA: 250uM), and stimulated for 48 h; 2.after 48 h, the drug-added medium was replaced with normal medium; 3.the Huc-MSCs cells were digested and counted, and the cells were spread in the co-culture upper chamber of group C at the amount of 2 × 10^6^ cells HucMSCs cells were digested and counted, and 2 × 10^6^ cells were spread on the C group transwell at a ratio of 1:2 between AML12 and HucMSCs; 4. Replaced the medium of group D with the configured 3 ml Huc-MSCs-Ex (400 μg/ml) solution; 5. Replace group E medium with 3 ml of Huc-MSCs supernatant solution and co-cultivate for 24–48 h, respectively, to detect the molecular or proteomic transfection efficiency for subsequent pathological staining, QP or WB assays (Fig. 1A).

### Construction of mouse NASH model and experimental intervention

The mice were randomly divided into 6 groups according to the culture period: (1) 6 weeks normal group, (2) 6 weeks HFMRCD model group, (3) 6 weeks HFMRCD + Ex treatment group, (4) 8 weeks normal group, (5) 8 weeks HFMRCD model group, (6) 8 weeks HFMRCD + Ex treatment group; Prior to the start of formal experiments, mice in the normal group were acclimated to normal chow, while mice in the model and treatment groups were fed a 1: 2 mixture of HFMRCD chow and normal chow on days 1–2; HFMRCD chow mixed with normal chow on days 3–4; HFMRCD chow mixed with normal chow on days 5–7; and HFMRCD chow mixed with normal chow on day 8, and until week 4, week 6, and week 8, the model group was fed HFMRCD and the blank group was fed normal chow during the experimental period. At weeks 4, 6, and 8, mice in the model group were fed HFMRCD; the blank group was fed normal chow during the experimental period.Starting at week 7 or 9, mice in the exosome-treated group received tail vein injections at the maximum tolerated dose of 2 mg/kg (40 µg/mL, once every 5 days) for one month (Fig. 1B)^39,41^.

After 12 h of fasting after the last injection, blood was collected by Neck dislocation method, weighed and recorded, the thoracic and abdominal cavities were opened, relevant morphological photographs were taken after releasing the liver, and serum and liver tissues were collected for relevant HE、Masson staining or molecular and proteomic transfection efficiency.The liver was then stained with HE staining to observe the structure of liver tissue and the degree of steatosis, and the degree of inflammatory cell infiltration and steatosis was scored and graded according to the NAFLD activity scoring (NAS) system^42^. The degree of steatosis was graded according to the ratio of area occupied by fat vacuolated degenerated cells/total cells into four grades such as 0, < 1/3, l/3–2/3 and > 2/3, the degree of inflammation (in terms of number of foci of necrosis) within the liver lobules into four grades such as 0, < 2, 2–4 and > 4, and the degree of hepatocellular ballooning was graded according to 0, few, most and /. Samples with a total score ≥ 5 were diagnosed as "NASH".

### Quantitative reverse transcription PCR (RT-qPCR)

Cells were rinsed three times with 2–3 mL of PBS buffer and 1 mL of TRIzol lysate (Life, Cat. No. 15596-018) was added, and total RNA was extracted according to the protocol described by the manufacturer and RT-qPCR was performed as described previously. Reverse transcription was performed using the SureScript™ First-Strand cDNA Synthesis Kit (GeneCopoeia, QP056), followed by BlazeTaq™ SYBR®Green Mix 2.0 (GeneCopoeia, QP031) and specific primers (DynaTech Biotechnology Ltd.) (Supplementary Table 3) for RT-qPCR reactions on a CFX96 quantitative real-time fluorescence PCR instrument (Thermo Fisher Scientific, TCR0096).Data and figure were analyzed using SDSV 2.4 software (Life Technologies).

### Western blotting analysis

This gel blot was cut in half for subsequent protein detection prior to antibody hybridization. Afterwards, Samples were run on 10% SDS-PAGE (Solarbio, S8010) and transferred to PVDF membrane (Millipore, K2MA8350E). Membranes were incubated with anti-TNF-α, anti-IL-6, anti-TGF-β1, anti-EI24, anti-AMPK, anti-mTOR, or anti-p62 and anti-LC3B (1: 500) overnight at 4 °C, and the membranes were applied to 1XTBST (Solarbio, 71080) and 5% skim milk (BD, 2271470), washed, incubated with enzyme-conjugated secondary antibody (1:5000 ratio), fully reacted with Immobilon Western HRP Substrate Luminol Reagent (Affinity. KF001) and automatically exposed in gel imaging (Monad. IP0521), and chemiluminescence signal intensity was quantified using imagelab software.

### Statistical analysis

Graphpad Prism 6.0 software (California, CA) was used for statistical analysis of the experimental data. The criterion for statistical significance of differences between means was *p* < 0.05.

### Equipment and settings

All column or trend statistical analysis graphs were captured and processed by Graphpad Prism 6.0 software (California, CA); while measured tissue staining images, such as oil red O or HE stained color pictures were first observed and scanned with an inverted microscope, and then uploaded and processed by Image-pro Plus 6.0 software; while Masson images were analyzed and processed by ImageJ software; all WB strip images were processed by gel imager with multiple exposures or using Imagelab software.

### ARRIVE guidelines

All authors confirmed the study is reported in accordance with ARRIVE guidelines.

### Supplementary Information

Supplementary Information.

## Data Availability

All data generated or analysed during this study are included in this published article [and its supplementary information files].
