# Supplementary material for: Comparison of effects of HucMSCs, exosomes, and conditioned medium on NASH
Source: Sci Rep. 2023 Oct 27;13:18431. doi: 10.1038/s41598-023-45828-3 (PMC10611740; doi:10.1038/s41598-023-45828-3)
Supplement: Supplementary file 1 — Supplementary Information. [file 41598_2023_45828_MOESM1_ESM.docx]

**Comparison of effects of HucMSCs, exosomes and conditioned medium on NASH**

Chenchen Liang, Siyuan Gao, Jianpeng Gao^*^, Yanwen XU, Qilong Li


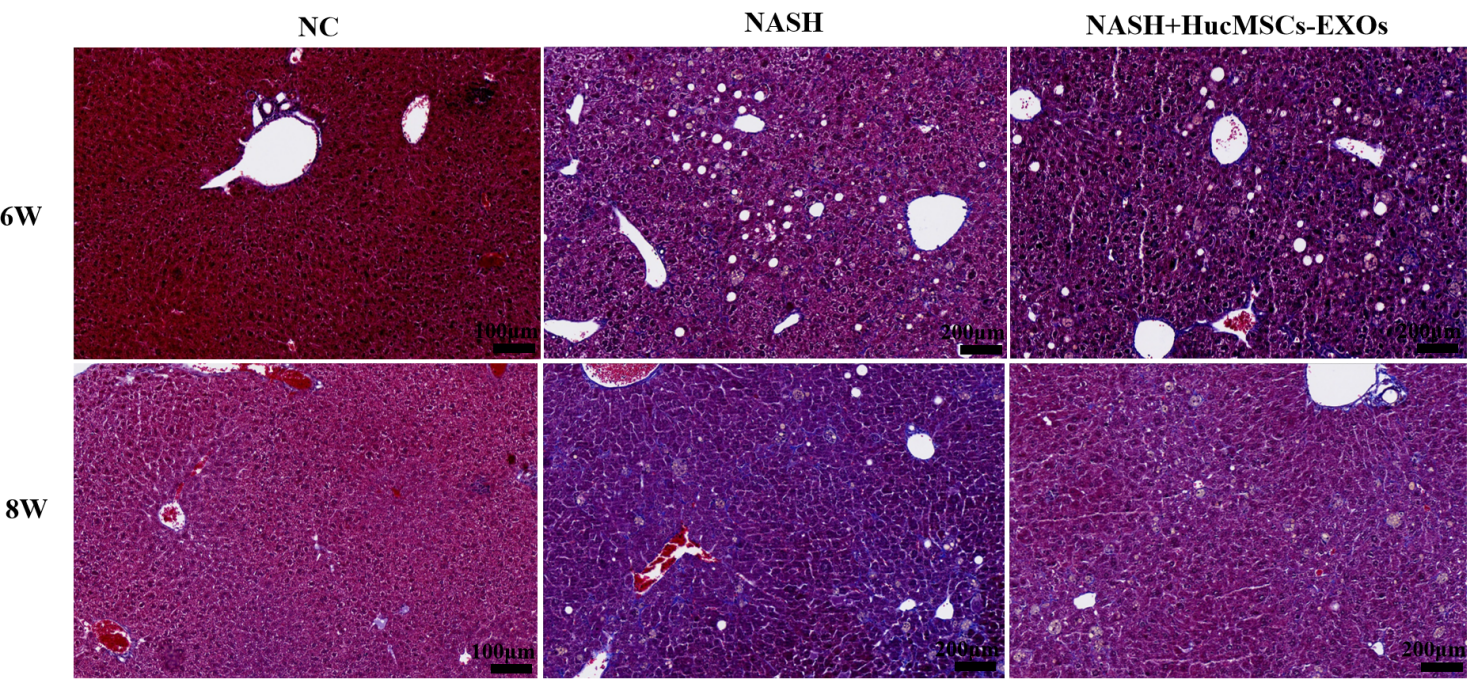
Supplementary Figure 1. Masson staining for collagen deposition in mouse liver tissue (400X)


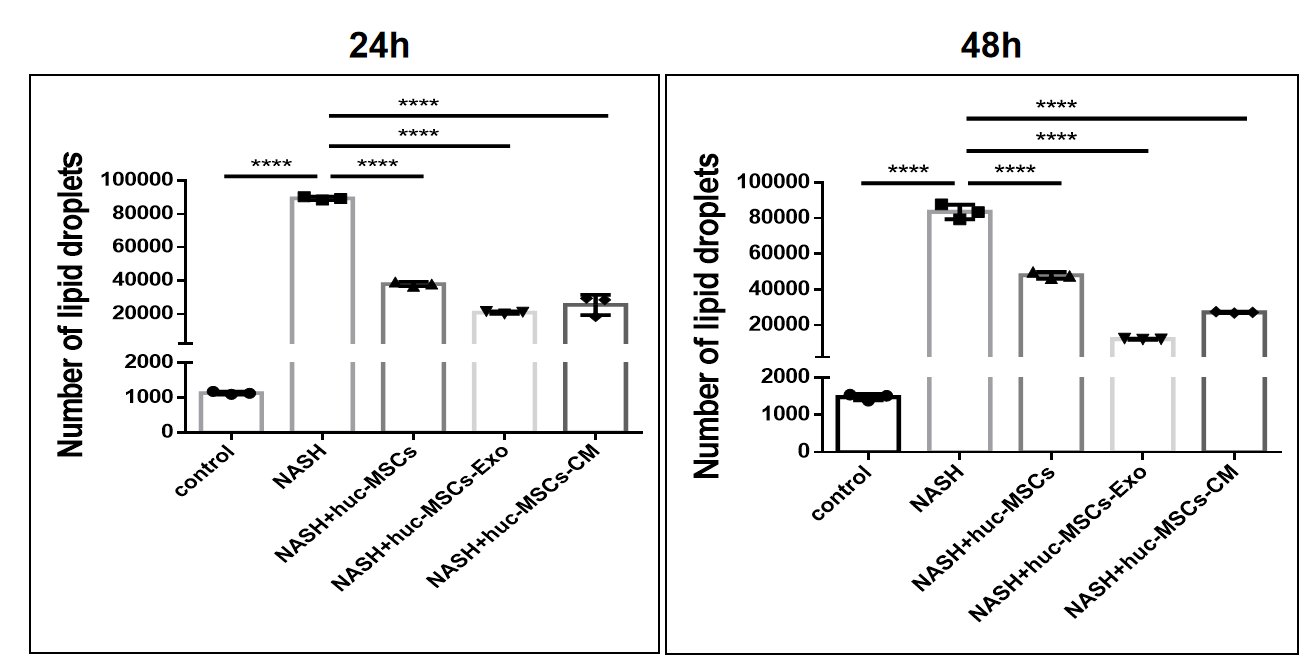
Supplementary Figure 2. Quantitative analysis of 24h or 48h cellular lipid droplets


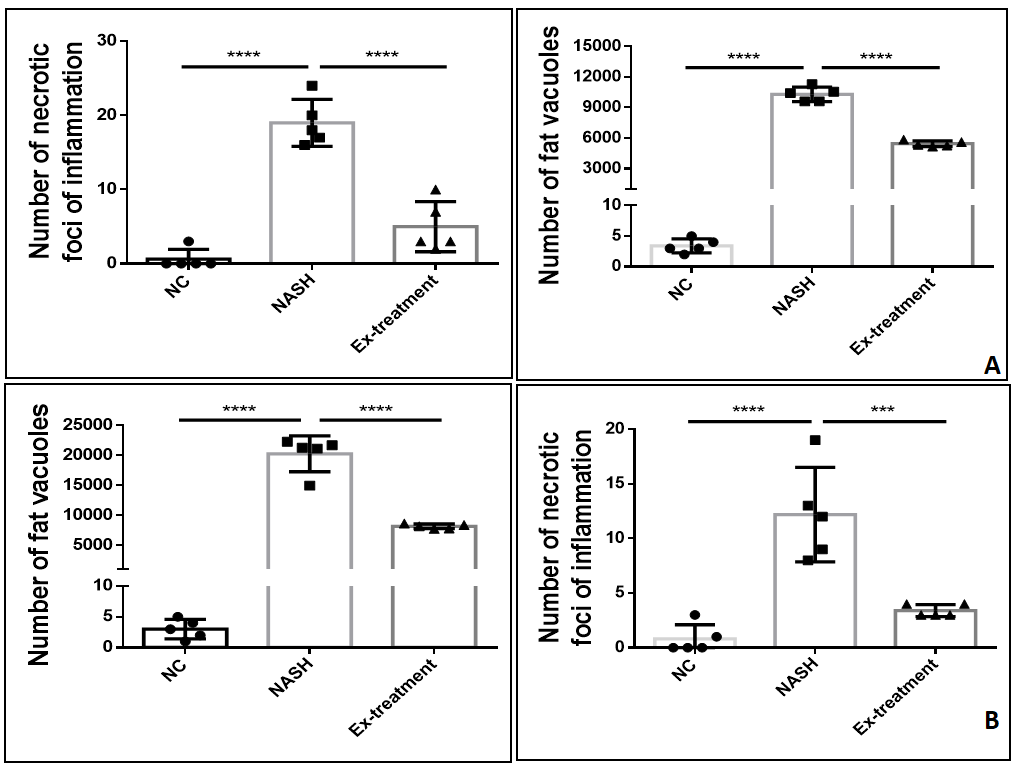
Supplementary Figure 3. Quantitative Analysis. A: Number of fat vacuoles at week 6 and 8; B: Number of foci of inflammatory necrosis at week 6 and 8


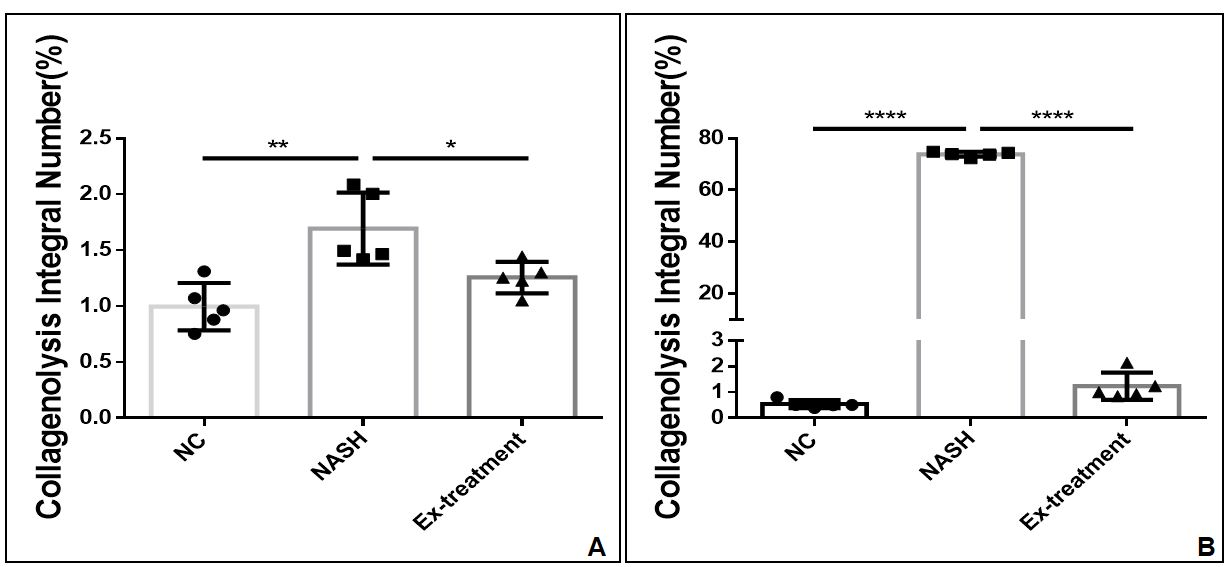
Supplementary Figure 4. Quantitative Analysis. A: Quantitative analysis of Masson collagen deposition at week 6; B:Quantitative analysis of Masson collagen deposition at week 8


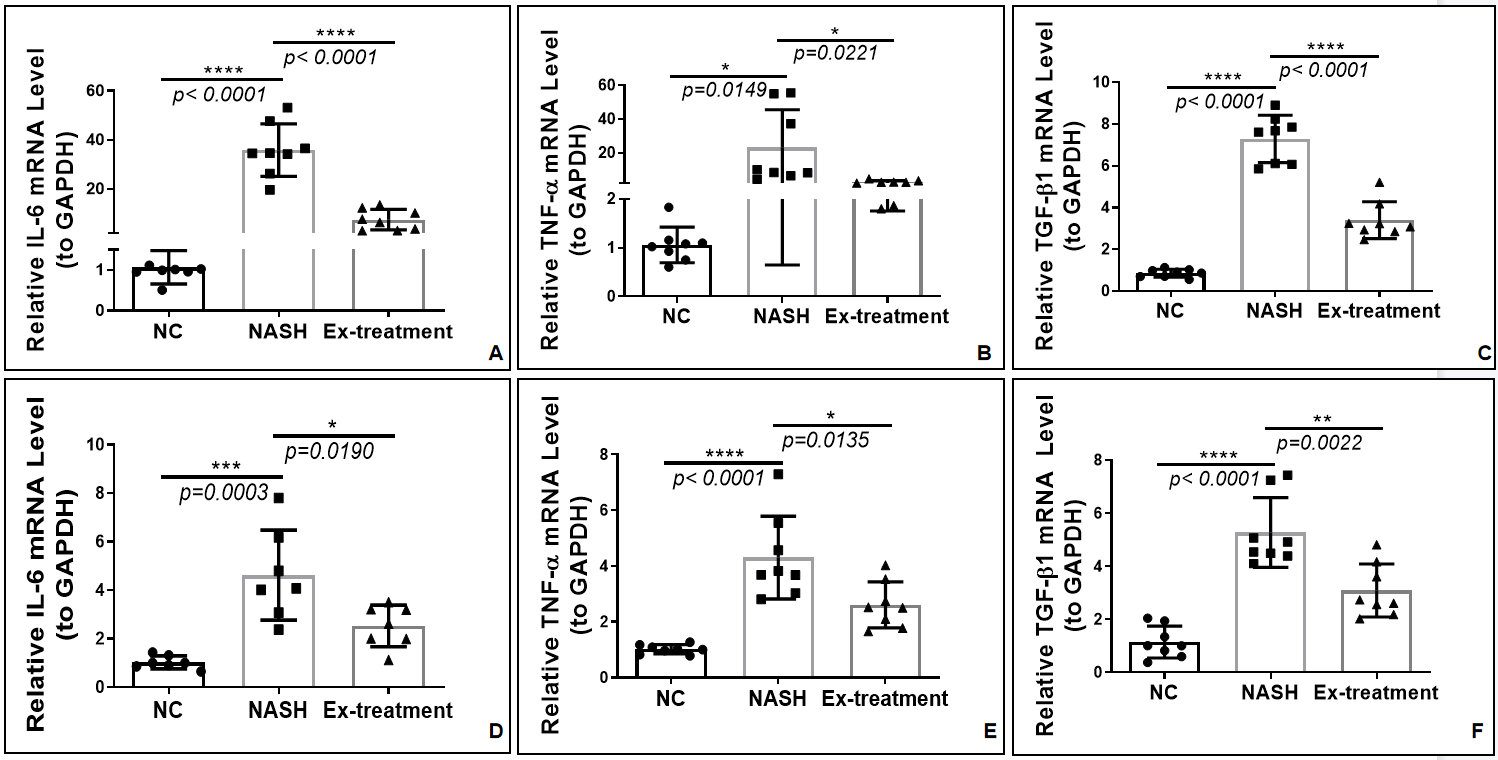
Supplementary Figure 5. A:6thW IL-6 mRNA expression levels (8 mice pooled); B:6thW TNF-α mRNA expression levels (8 mice pooled); C:6thW TGF-β1 mRNA expression levels (8 mice pooled); D:8thW IL-6 mRNA expression levels (8 mice pooled); E:TNF-α mRNA expression levels at 8thW (8 mice pooled); F:8thW TGF-β1 mRNA expression levels (8 mice pooled)


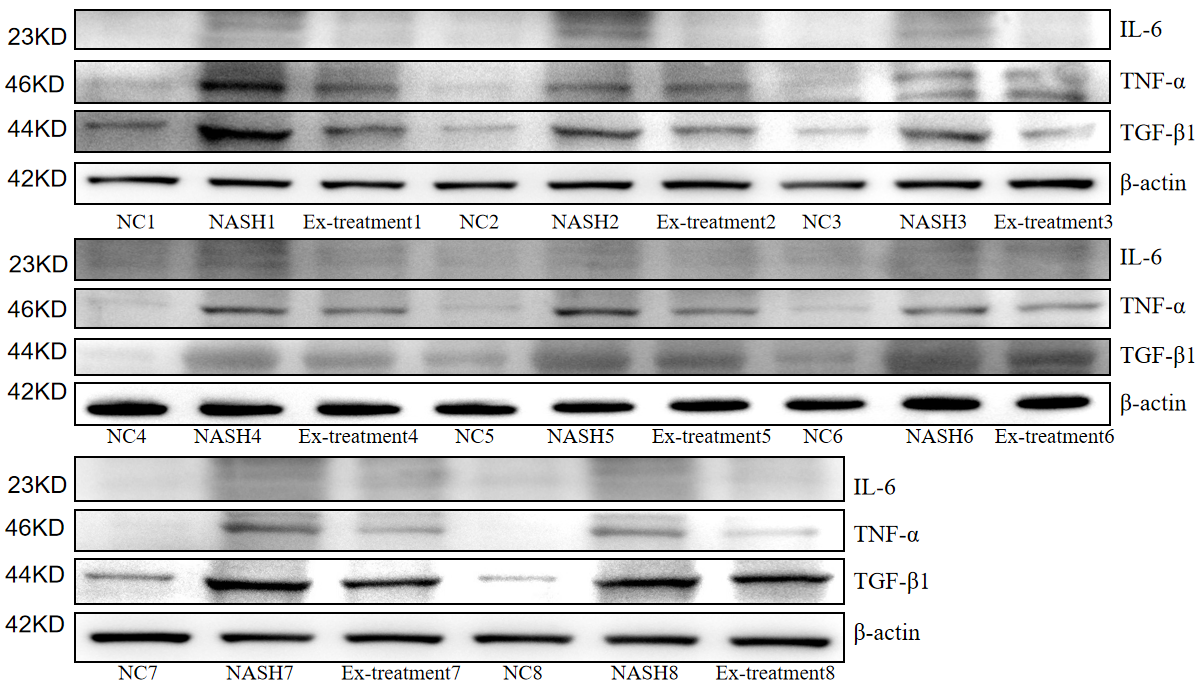
Supplementary Figure 6 . 6thW IL-6, TNF-α, TGF-β1 protein immunoblot(8 mice level) (Note: This gel blot was cut in half for subsequent protein detection prior to antibody hybridization. IL-6, TNF-α, TGF-β1 and β-actin groups using multiple exposures; and blots or gels have been cropped, the IL-6, TNF-α, TGF-β1 and β-actin original blots or gels are shown in the Supplementary Figure 29)


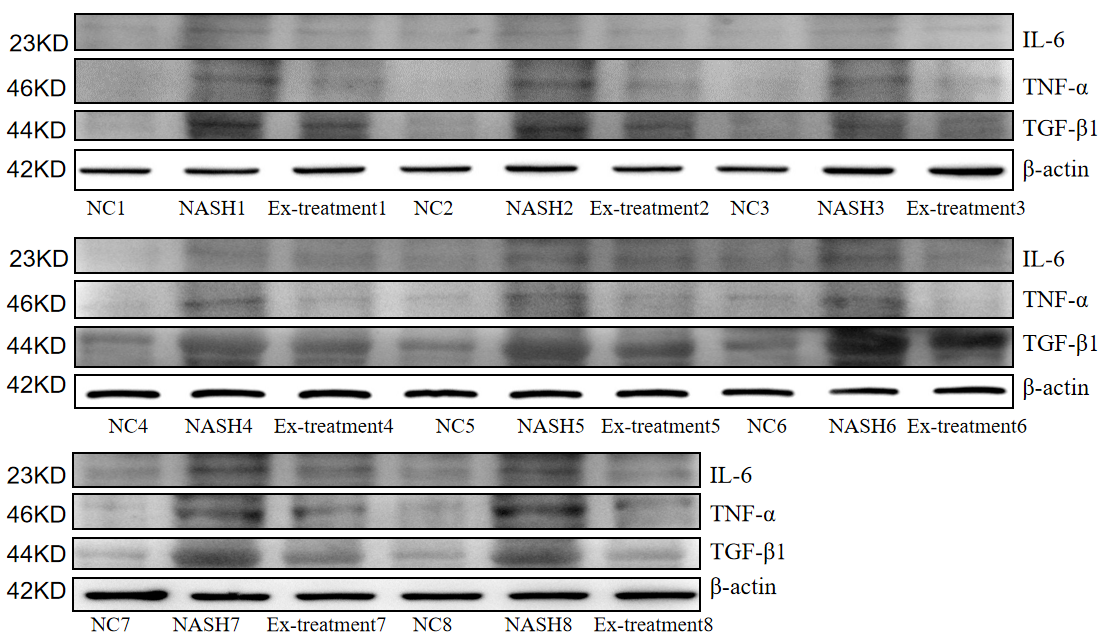
Supplementary Figure7. 8thW IL-6, TNF-α, TGF-β1 protein immunoblot levels

(8 mice level) (Note: This gel blot was cut in half for subsequent protein detection prior to antibody hybridization. IL-6, TNF-α, TGF-β1 and β-actin groups using multiple exposures; and blots or gels have been cropped, the IL-6, TNF-α, TGF-β1 and β-actin original blots or gels are shown in the Supplementary Figure 30)


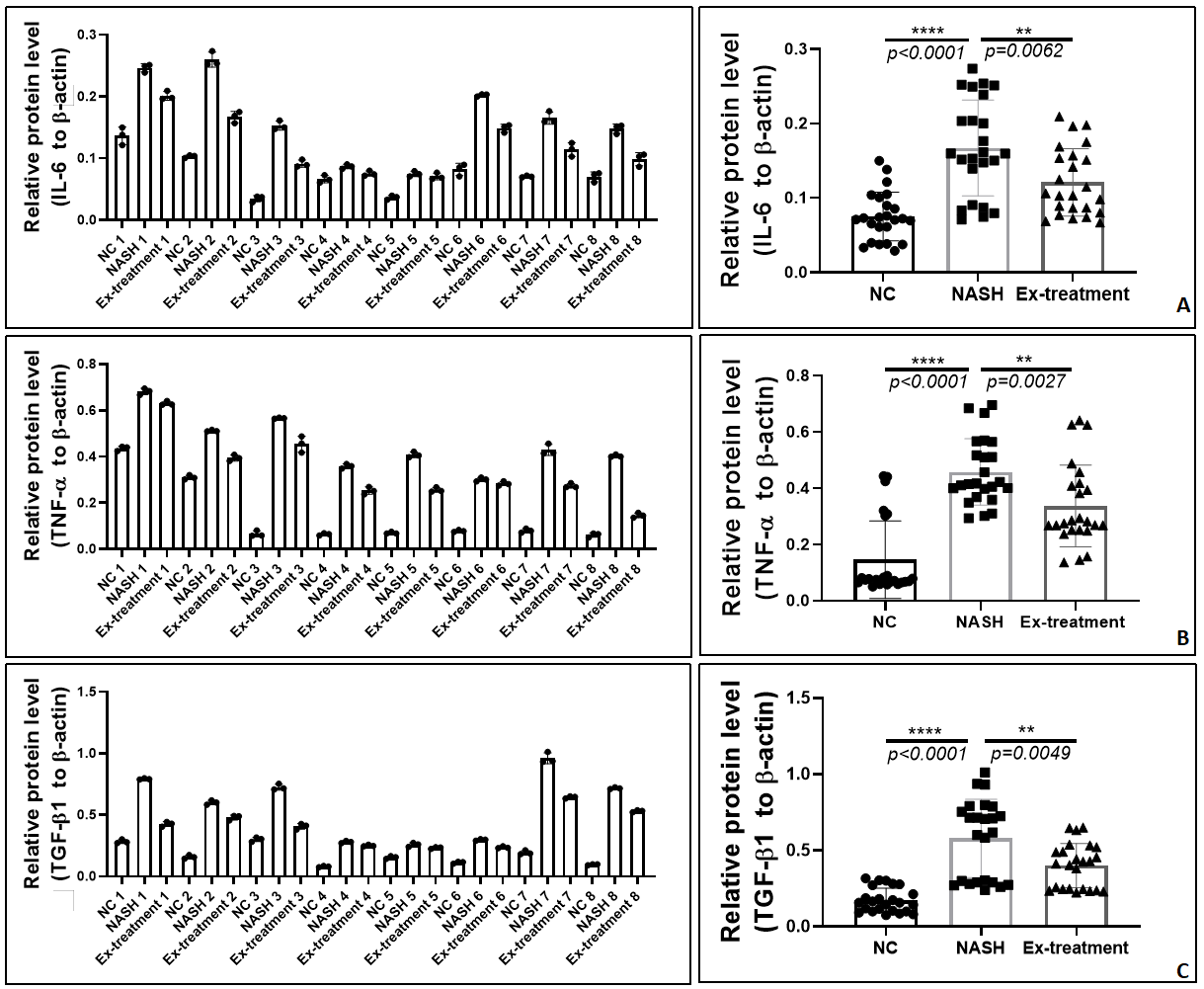


Supplementary Figure 8. A: 6thW IL-6 protein expression levels (8 mice pooled); B: TNF-α protein expression levels at 6thW (8 mice pooled); C: 6thW TGF-β1 protein expression levels (8 mice pooled)


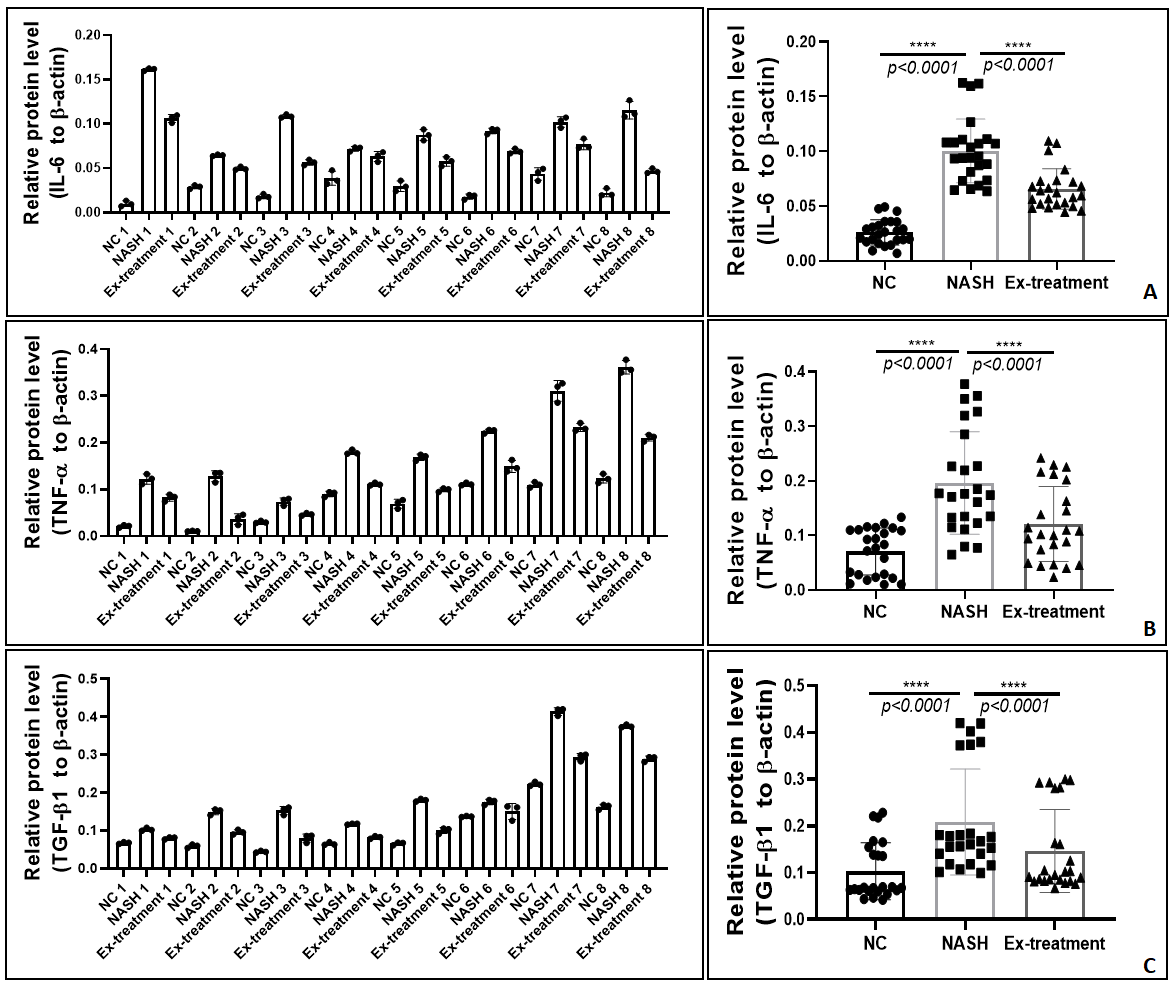
Supplementary Figure 9. A:8thW IL-6 protein expression levels (8 mice pooled); B: 8thW TGF-β1 protein expression levels (8 mice pooled); C: 8thW TNF-α protein expression levels (8 mice pooled)


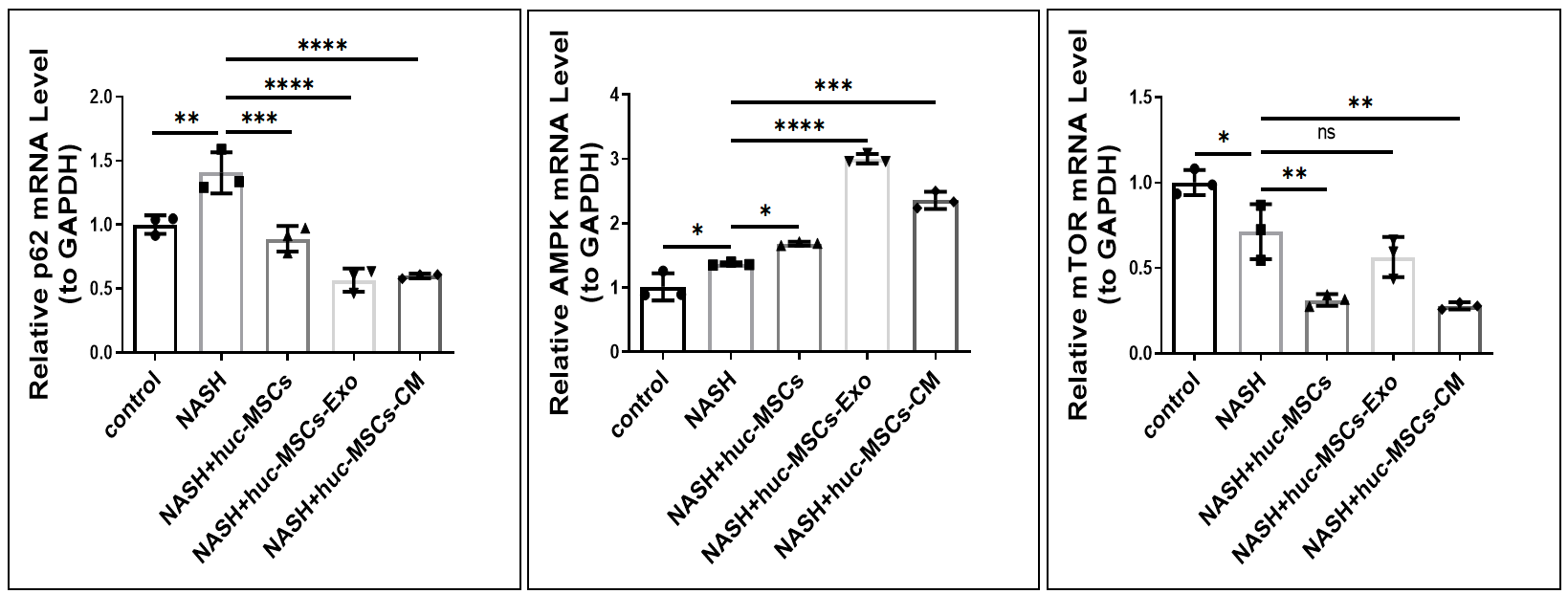
Supplementary Figure 10. P62, AMPK, mTOR mRNA levels (cellular level summary)


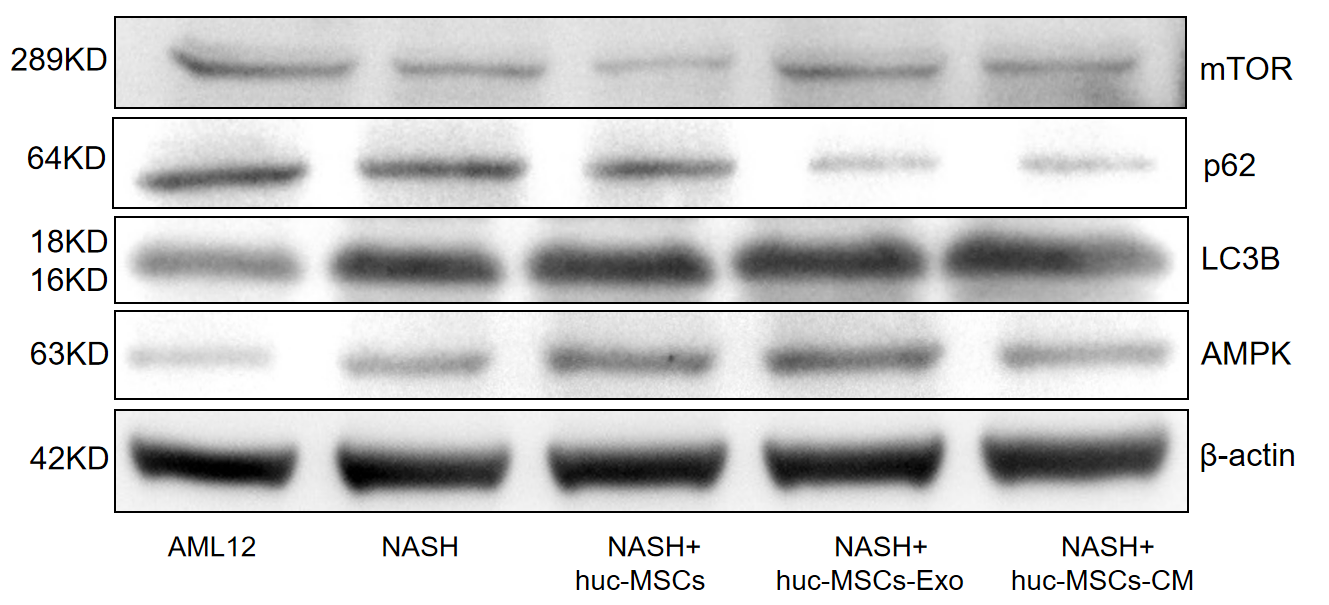


Supplementary Figure 11. Immunoblot levels of P62, AMPK, mTOR and LC3B proteins(cellular level summary) (Note: This gel blot was cut in half for subsequent protein detection prior to antibody hybridization. P62, AMPK, mTOR and LC3B groups using multiple exposures; and blots or gels have been cropped, the P62, AMPK, mTOR and LC3B original blots or gels are shown in the Supplementary Figure 31)


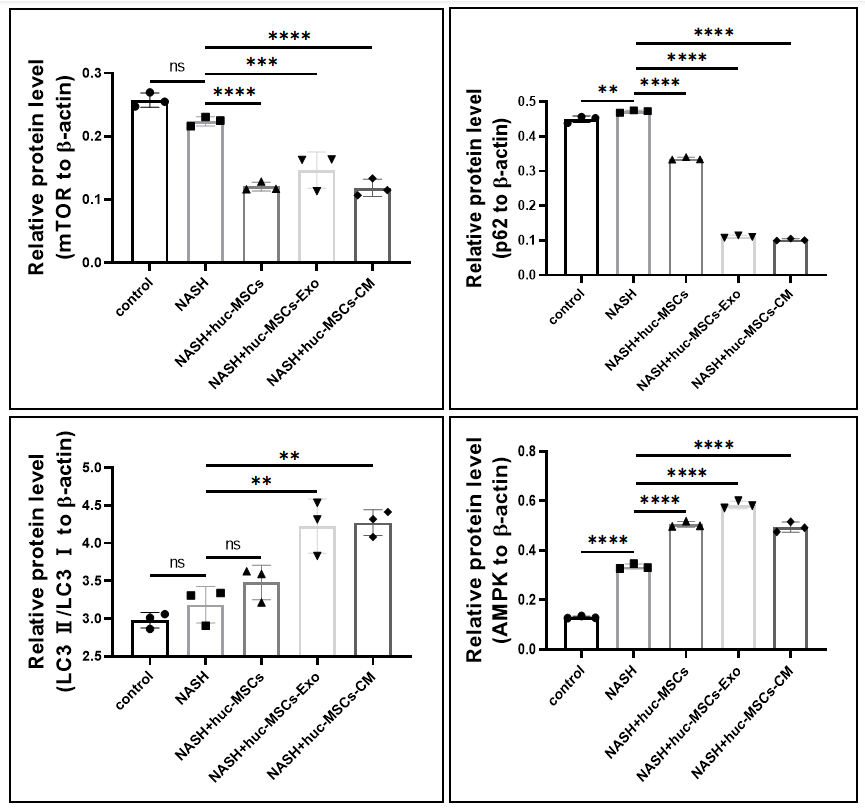
Supplementary Figure 12. P62, AMPK, mTOR, LC3B protein expression levels levels (cellular level summary)


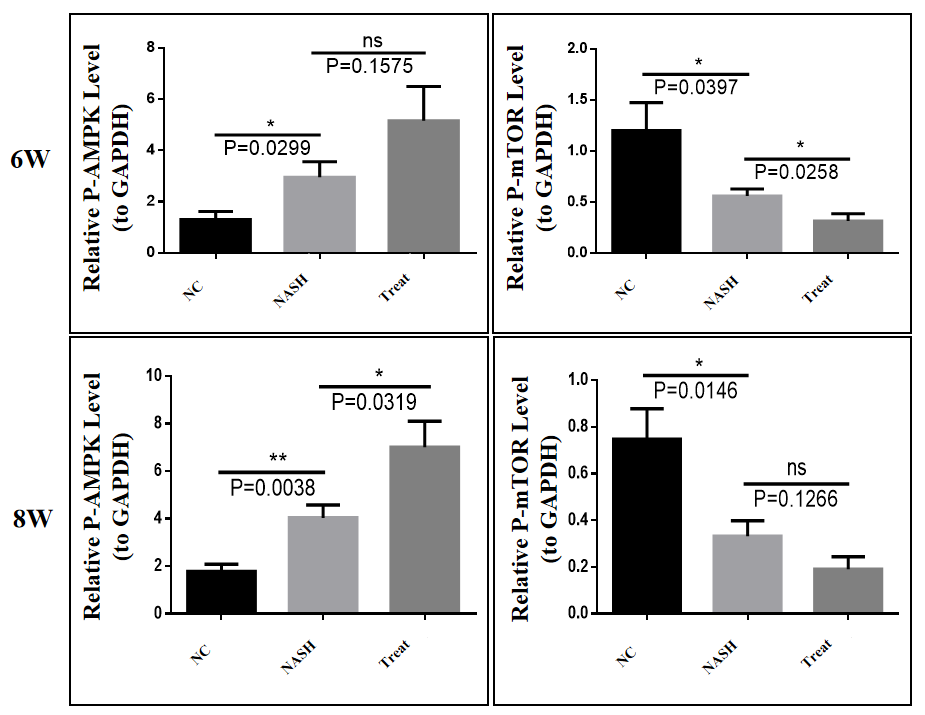
Supplementary Figure 13 . P-AMPK, P-mTOR expression levels at 6thW and 8thW


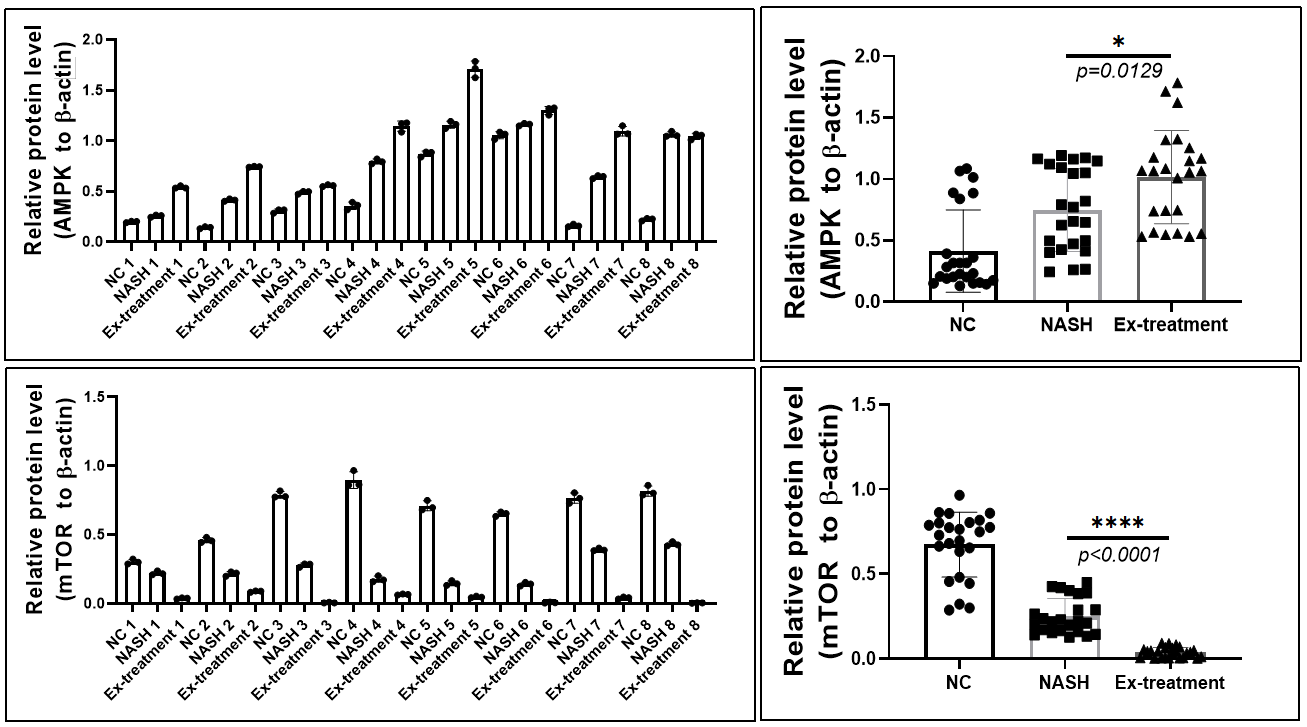
Supplementary Figure14. A: Levels of AMPK protein expression in the 6thW; B:Levels of mTOR protein expression in the 6thW


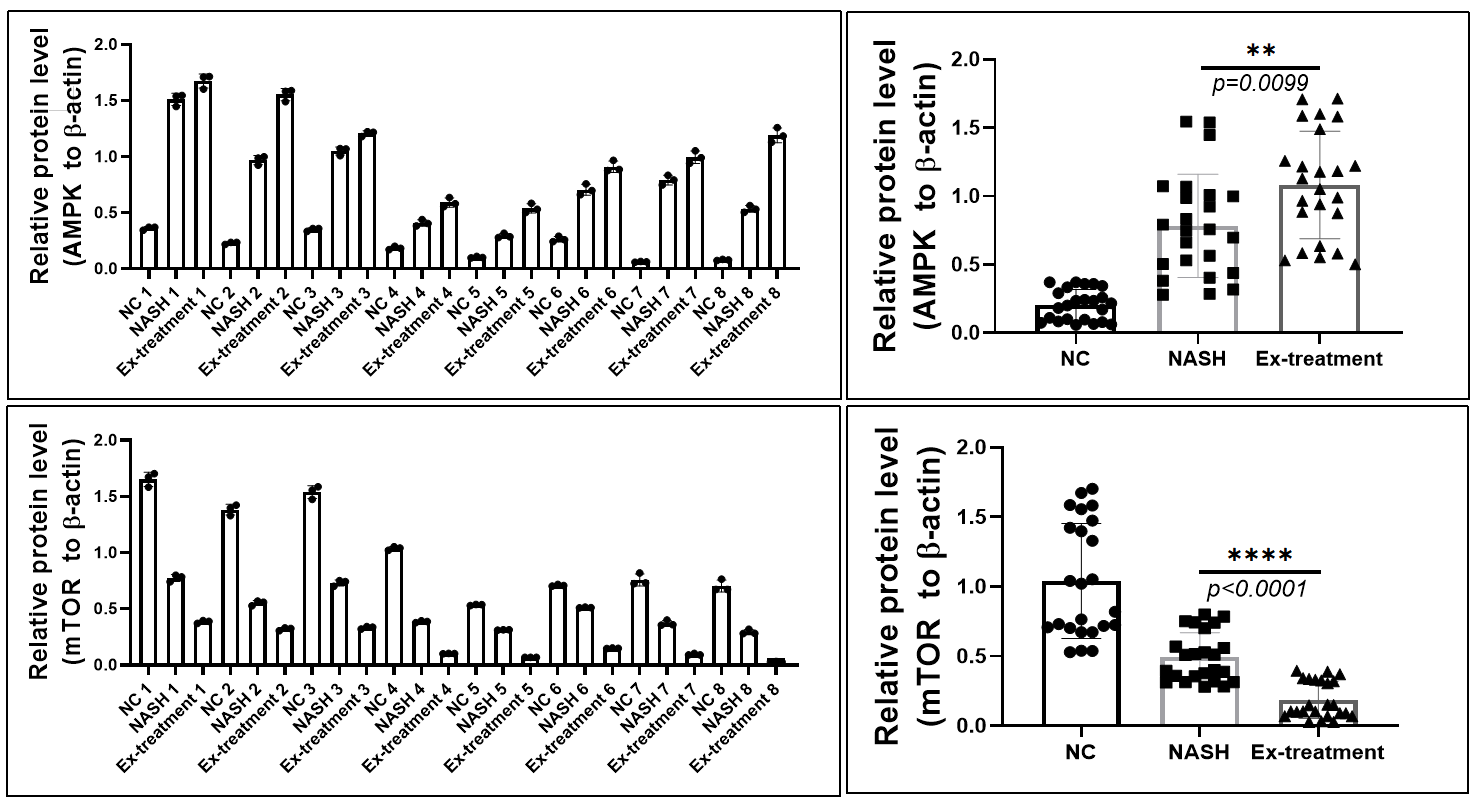
Supplementary Figure15. A:Levels of AMPK protein expression in the 8thW; B:Levels of mTOR protein expression in the 8thW


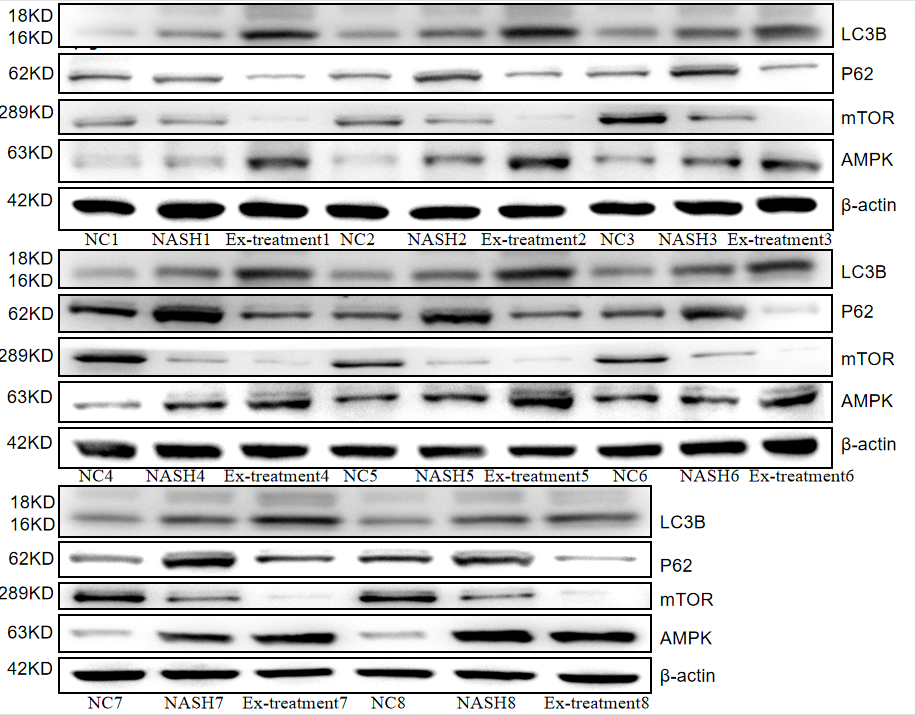
Supplementary Figure16. 6thW P62, AMPK, mTOR, LC3protein immunoblot levels

(8 mice level) (Note:This gel blot was cut in half for subsequent protein detection prior to antibody hybridization. LC3B, P62, mTOR and AMPK groups using multiple exposures; and blots or gels have been cropped, the LC3B, P62, mTOR and AMPK original blots or gels are shown in the Supplementary Figure 32)


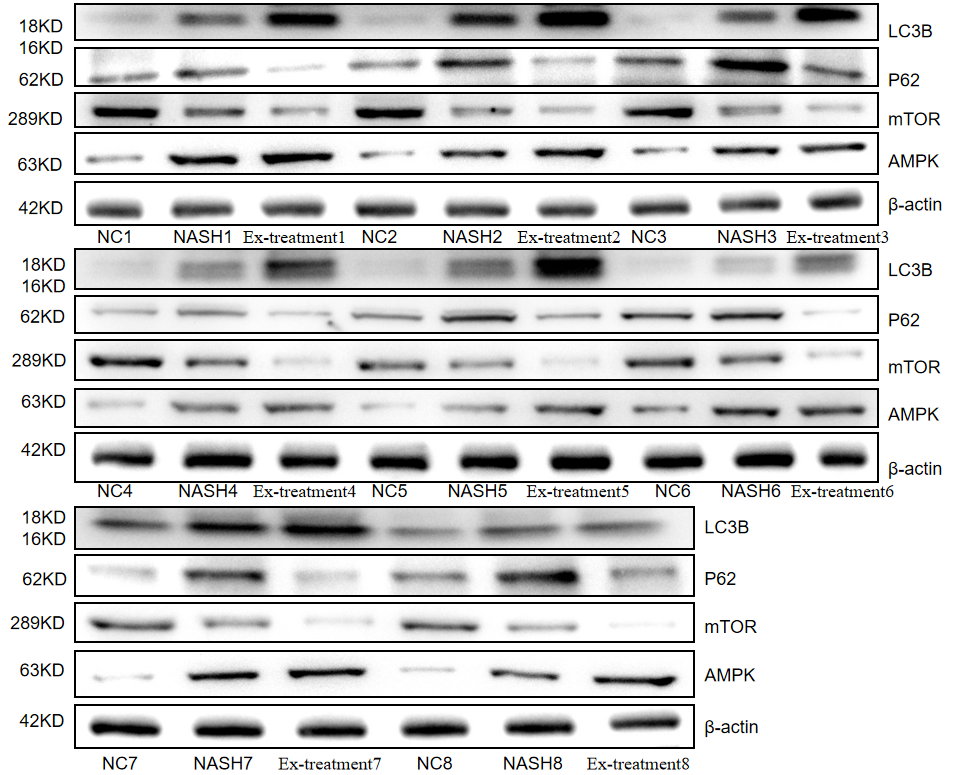
Supplementary Figure17. 8thW P62, AMPK, mTOR, LC3B protein immunoblot levels (8 mice level) (Note: This gel blot was cut in half for subsequent protein detection prior to antibody hybridization. LC3B, P62, mTOR and AMPK groups using multiple exposures; and blots or gels have been cropped, the LC3B, P62, mTOR and AMPK original blots or gels are shown in the Supplementary Figure 33)


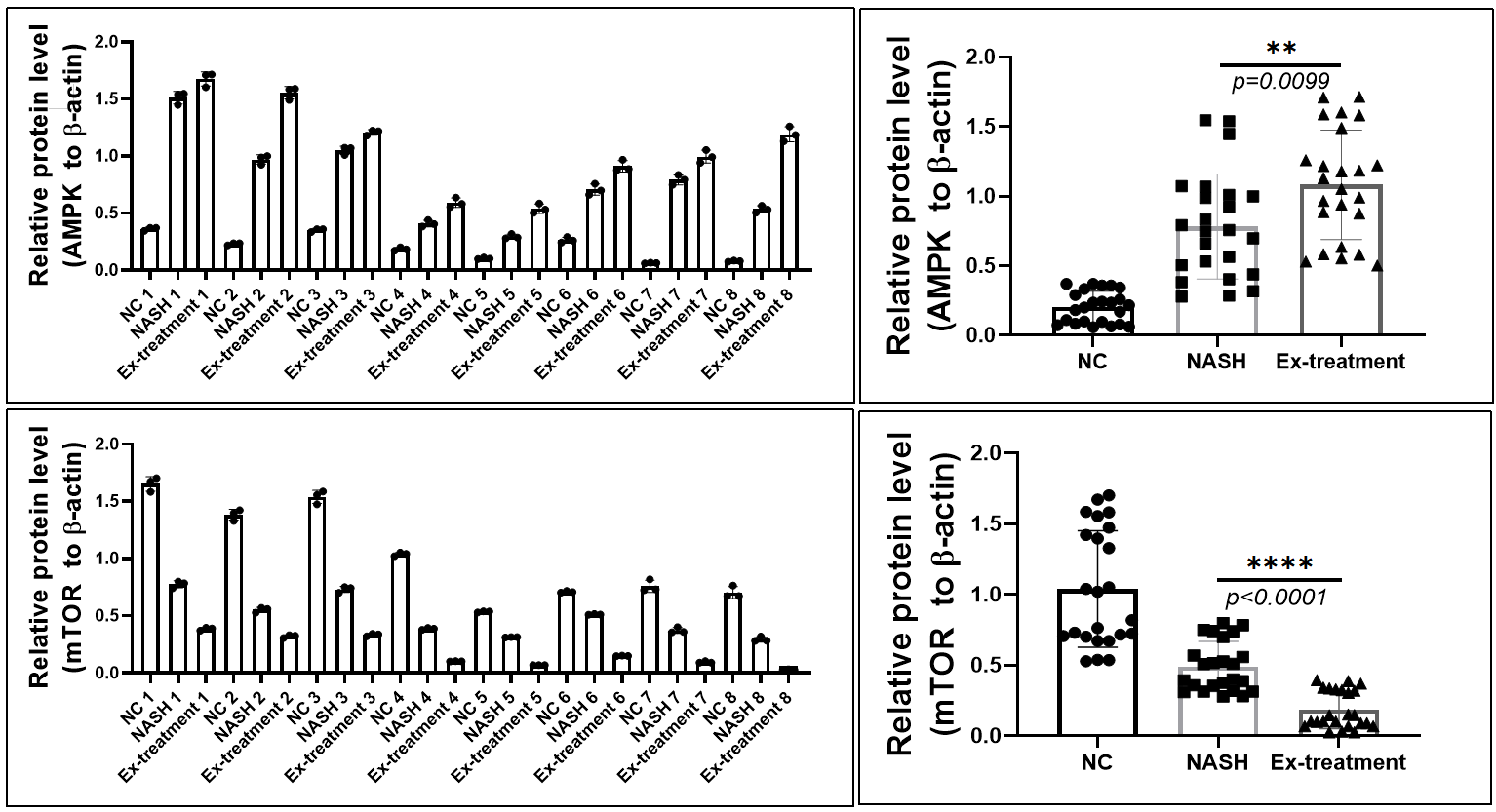
Supplementary Figure18. A：Levels of LC3BⅡ/Ⅰ protein expression in the 6thW；

B：Levels of P62 protein expression in the 6thW


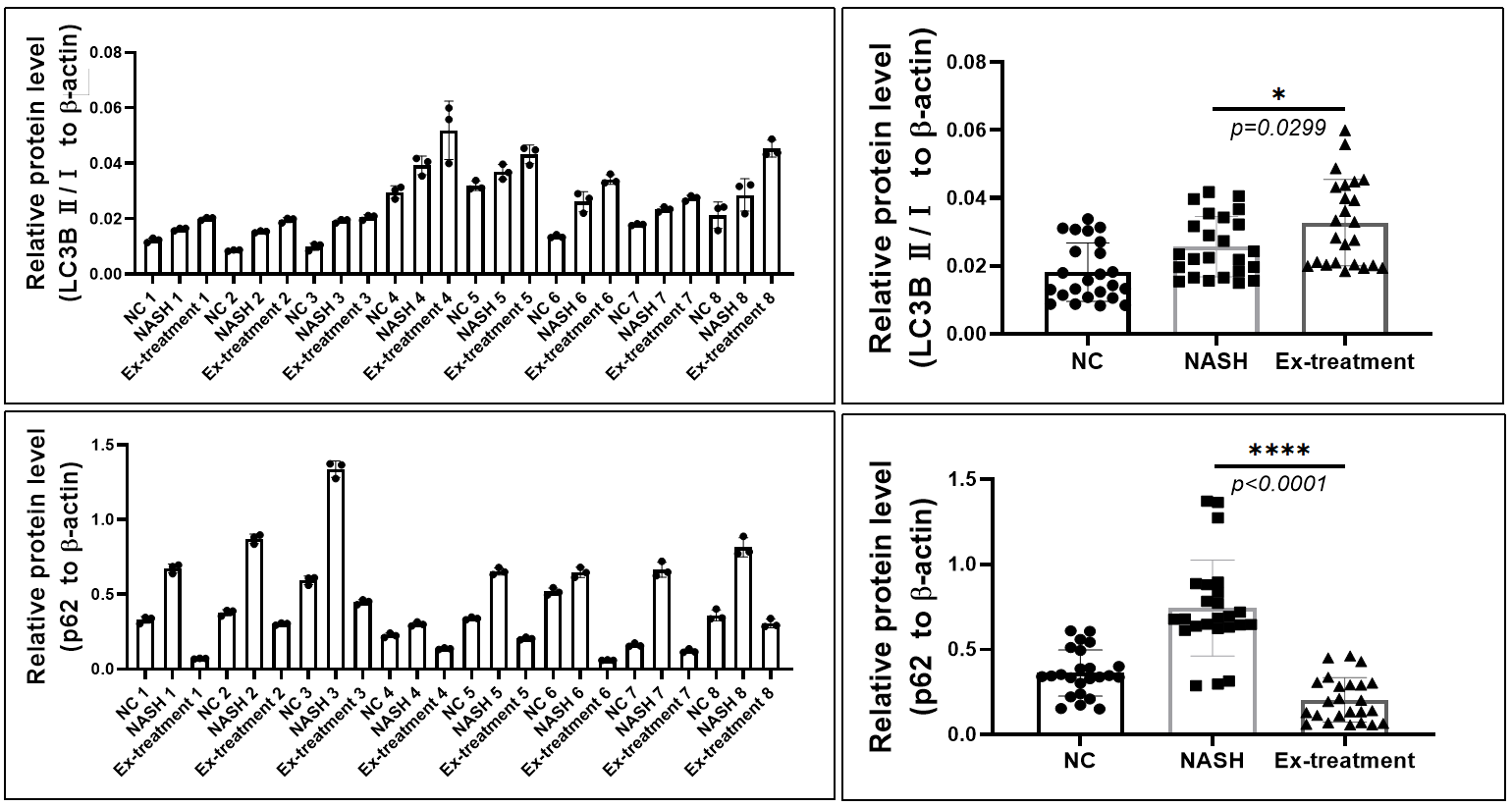
Supplementary Figure19. C：Levels of LC3BⅡ/Ⅰ protein expression in the 8thW；D：Levels of P62 protein expression in the 8thW


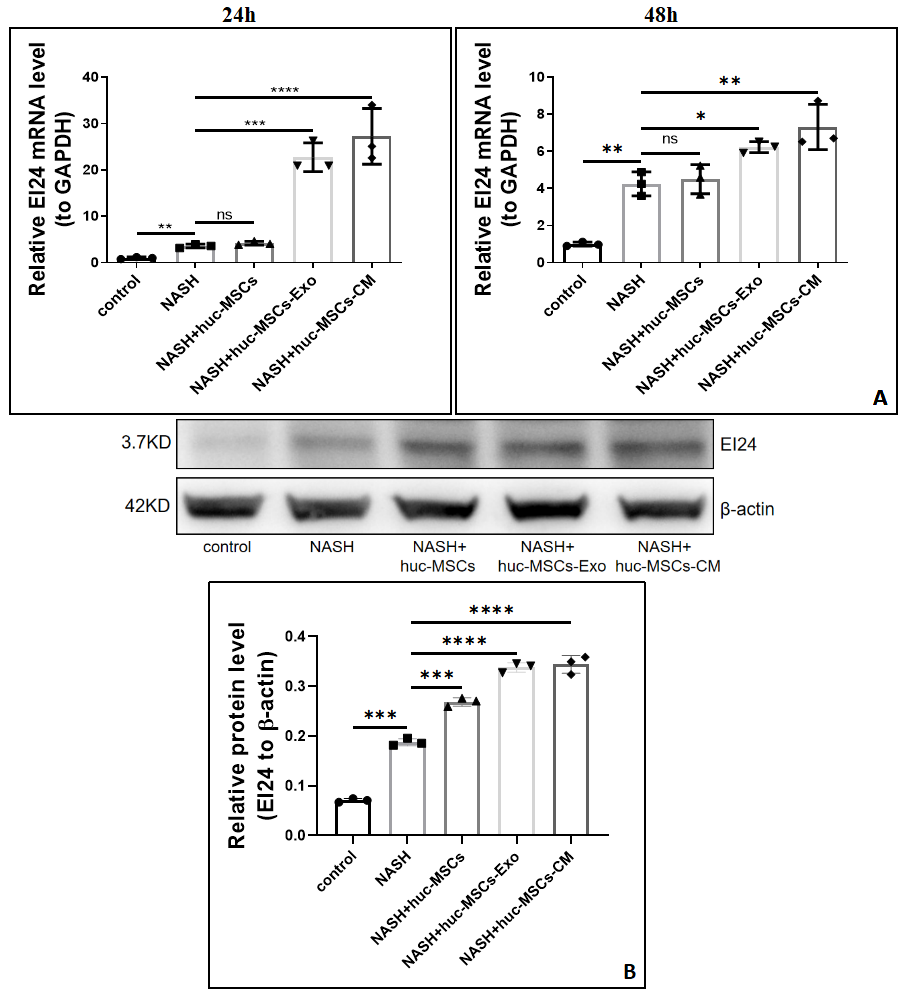
Supplementary Figure20. A：EI24 mRNA level (24-48h) (cellular level)；B：EI24 48h protein expression level (cellular level) (Note: This gel blot was cut in half for subsequent protein detection prior to antibody hybridization. EI24 group using multiple exposures; and blots or gels have been cropped, the EI24 original blots or gels are shown in the Supplementary Figure 34)


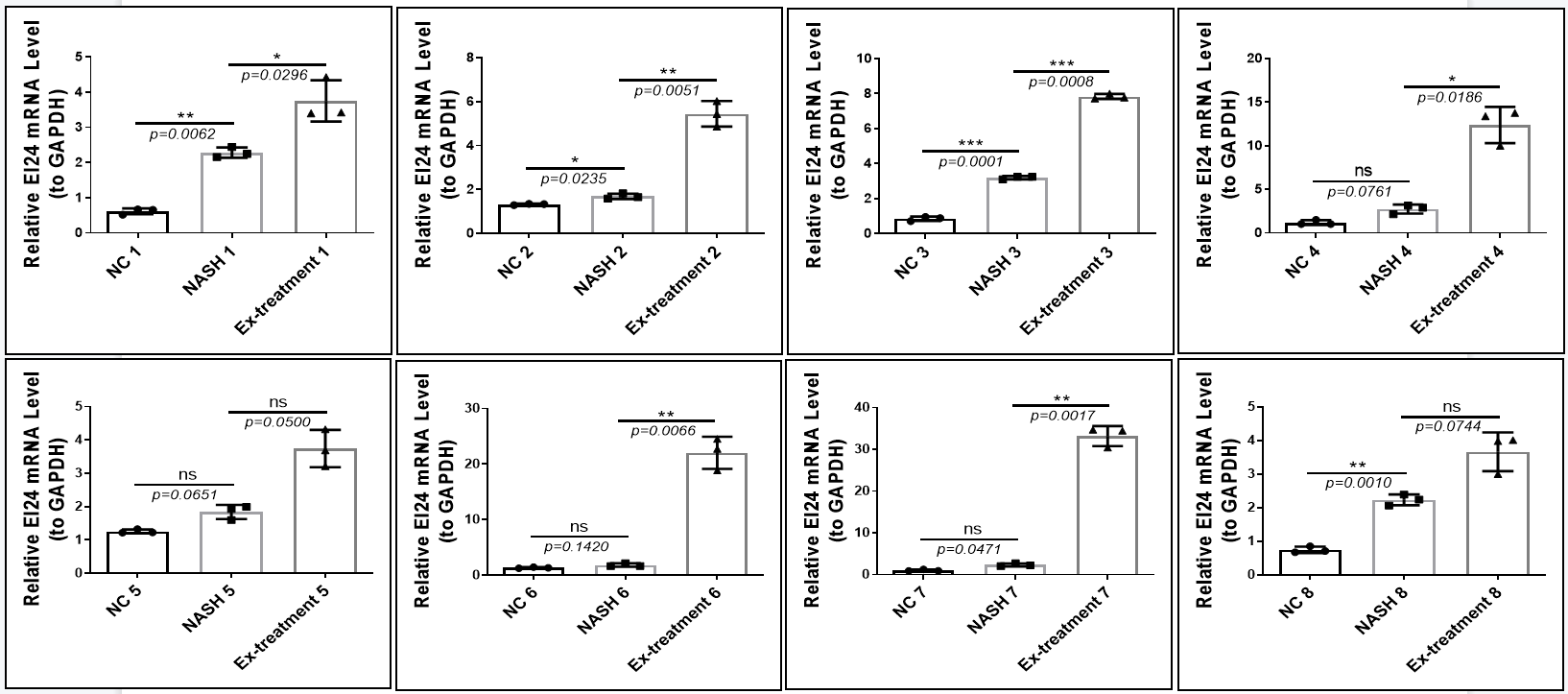
Supplementary Figure21. 6thW EI24 mRNA level


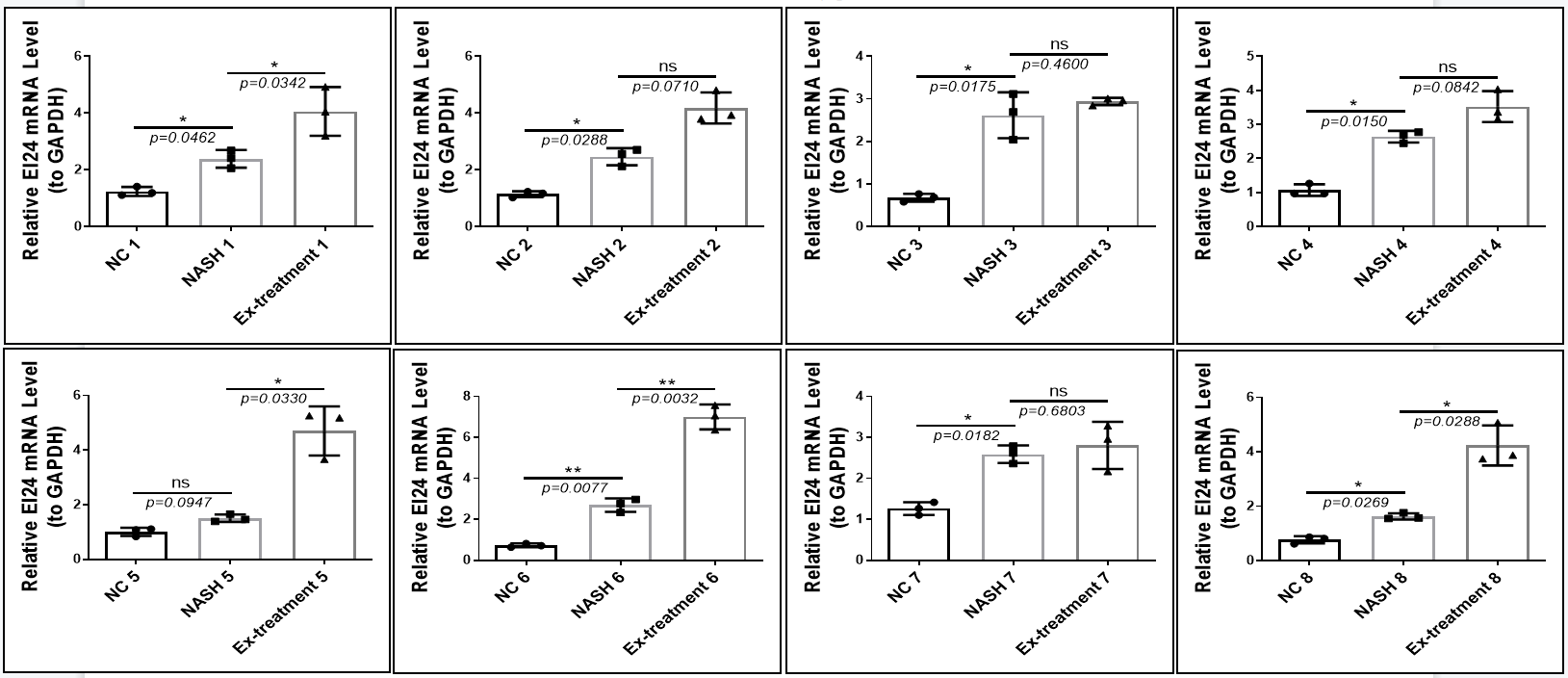
Supplementary Figure22. 8thW EI24 mRNA level


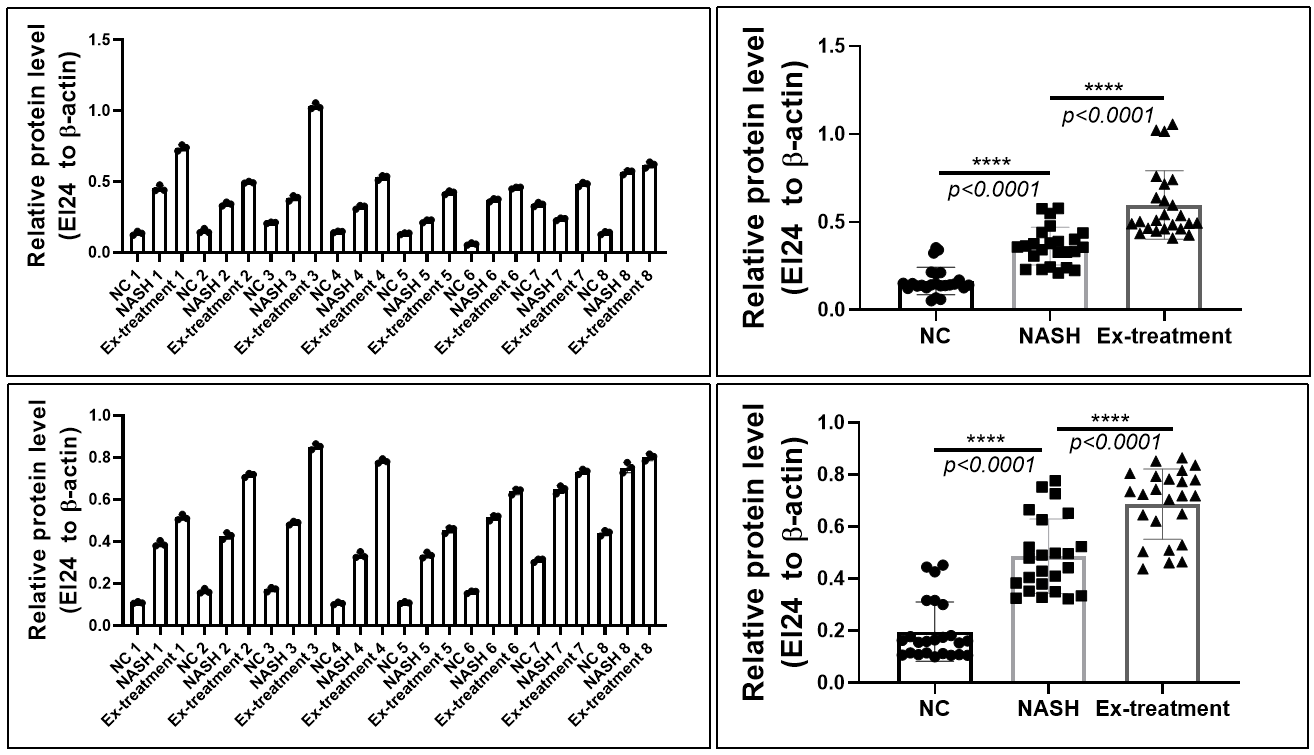
Supplementary Figure23. 6thW EI24 protein level；B：8thW EI24 protein level；


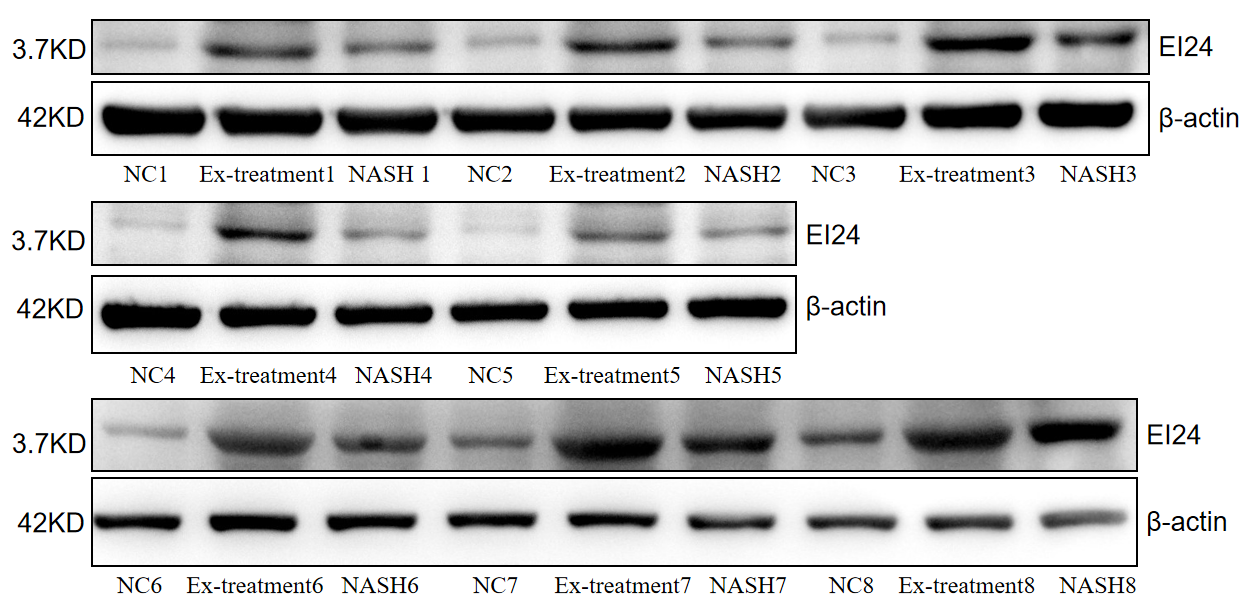
Supplementary Figure24. 6thW EI24 protein immunoblot levels(8 mice level) (Note: This gel blot was cut in half for subsequent protein detection prior to antibody hybridization. EI24 group using multiple exposures; and blots or gels have been cropped, the EI24 original blots or gels are shown in the Supplementary Figure 35)


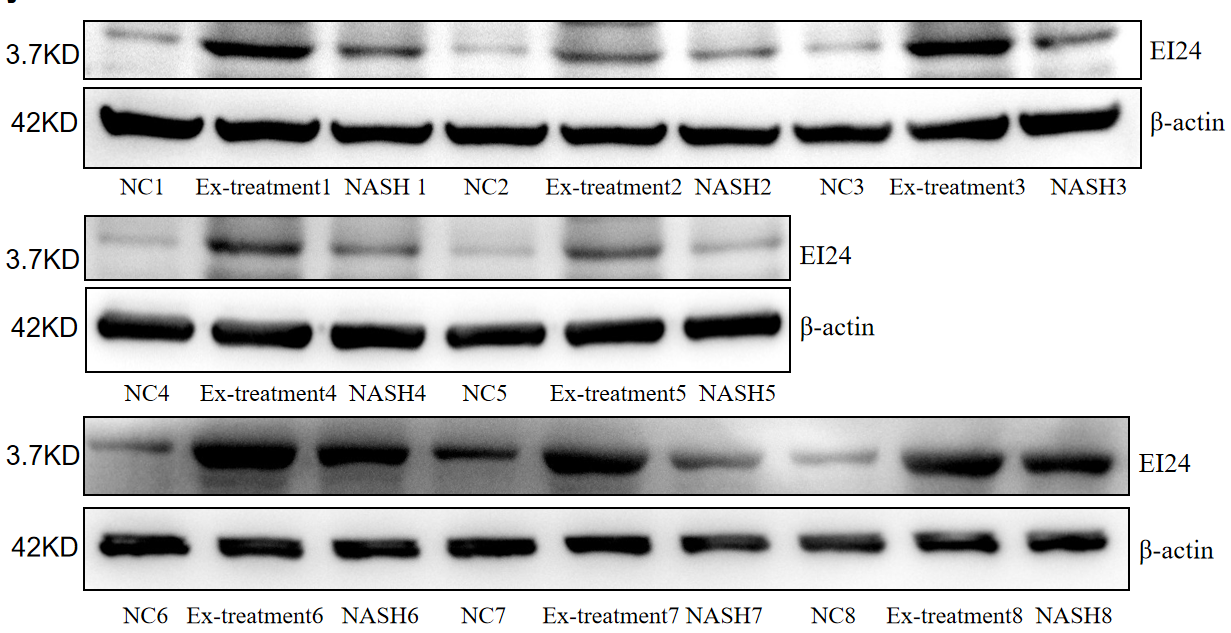
Supplementary Figure25. 8thW EI24 protein immunoblot levels(8 mice level) (Note:This gel blot was cut in half for subsequent protein detection prior to antibody hybridization. EI24 group using multiple exposures; and blots or gels have been cropped, the EI24 original blots or gels are shown in the Supplementary Figure 36)


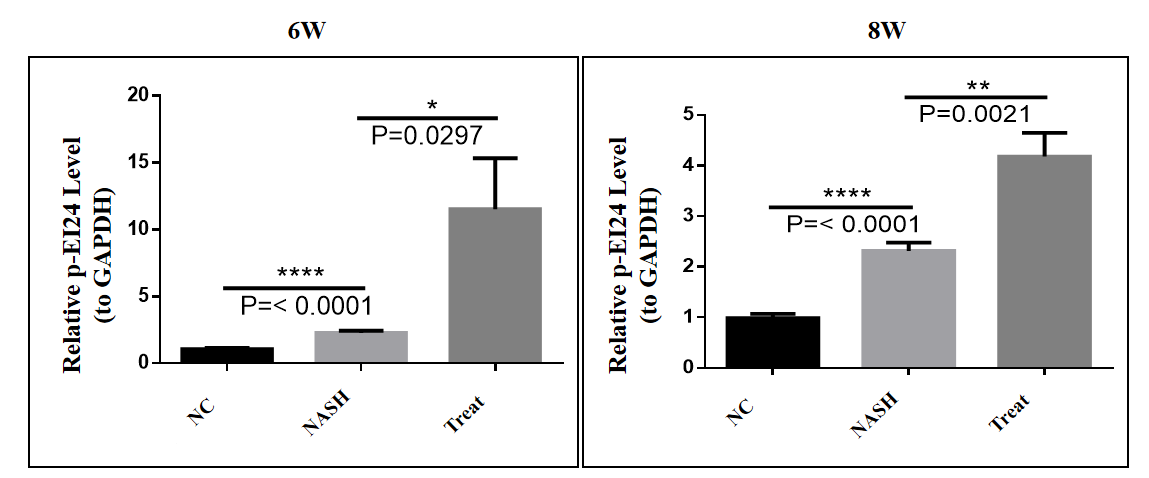


Supplementary Figure26. p-EI24 expression levels at 6thW and 8thW

Supplementary Table 1. Cross-reference to reagent spiki

| **96-well plate comparison table** | | | | | | | | | |
| --- | --- | --- | --- | --- | --- | --- | --- | --- | --- |
| / | 2 | 3 | 4 | 5 | 6 | 7 | 8 | 9 | 10 |
| B | Blank group | Cell group | Solvent group | 2000uM  OA  +  1000uM  PA | 1000uM  OA  +  500uM  PA | 500uM  OA  +  250uM  PA | 250uM  OA  +  125uM  PA | 125uM  OA  +  62.5uM  PA | 62.5uM  OA  +  31.25uM  PA |
| C |  |  |  |  |  |  |  |  |  |
| D |  |  |  |  |  |  |  |  |  |
| E |  |  |  |  |  |  |  |  |  |
| F |  |  |  |  |  |  |  |  |  |

Supplementary Table 2. Comparison of HucMSCs-Ex treated liver tissue steatosis, inflammation and ballooning pathology scores (x̅±s,n=8)

| Time | Groups | Animals | Steatosis | Inflammation | Ballooning  Change | Overall  Score |
| --- | --- | --- | --- | --- | --- | --- |
| 6 week | NC | *C57BL/6*Mice | 0 | 0 | 0 | 0 |
|  | NASH | *C57BL/6*Mice | 3.00±0.00 | 3.00±0.00 | 0.40±0.49 | 6.40±0.49 |
|  | Treatment | *C57BL/6*Mice | 0.80±0.40**** | 1.60±0.49*** | 0 | 2.40±0.80**** |
| 8 week | NC | *C57BL/6*Mice | 0 | 0 | 0 | 0 |
|  | NASH | *C57BL/6*Mice | 3.00±0.00 | 3.00±0.00 | 1.00±0.00 | 7.00±0.00 |
|  | Treatment | *C57BL/6*Mice | 0.60±0.49**** | 2.20±0.40** | 0 | 2.80±0.40**** |

Note: *（*p*<0.05）, **（*p*<0.01）, ***（*p*<0.001）, ****（*p*<0.0001）

Supplementary Table 3. The primers were made by DynaTech Biotechnology Ltd. and the sequences are as follows

| Name | Order | Base Number | Genus |
| --- | --- | --- | --- |
| M-IL-6-F | GAGACTTCACAGAGGATACCACTC | 24 | Mouse |
| M-IL-6-R | TGCCATTGCACAACTCTTTTCTC | 23 | Mouse |
| M-TNF-α-F1 | CACCACGCTCTTCTGTCTAC | 20 | Mouse |
| M-TNF-α-R1 | ACTTGGTGGTTTGTGAGTGT | 20 | Mouse |
| M-TGF-β1-F | CCGCAACAACGCCATCTA | 18 | Mouse |
| M-TGF-β1-R | GCCCTGTATTCCGTCTCCTT | 20 | Mouse |
| Ei24-F | GGGCATCTGTACCATCTCAA | 20 | Mouse |
| Ei24-R | AATACTCCACCATTCCAAGC | 20 | Mouse |
| M-Gapdh-F | TGCCCAGAACATCATCCCT | 19 | Mouse |
| M-Gapdh-R | GGTCCTCAGTGTAGCCCAAG | 20 | Mouse |

Supplementary Table 4. Analysis of changes in body weight growth rate

of mice at 6W

|  | 7w | | | | 10w | | | |
| --- | --- | --- | --- | --- | --- | --- | --- | --- |
|  | NC | NASH | Treatment | | NC | | NASH | Treatment |
| Average weight | 25 | 27 | 24 | | 25 | | 28 | 27 |
|  | 25 | 29 | 25 | | 27 | | 29 | 29 |
|  | 24 | 27 | 27 | | 26 | | 29 | 28 |
|  | 24 | 28 | 27 | | 25 | | 29 | 27 |
|  | 26 | 28 | 25 | | 27 | | 31 | 29 |
|  | 24 | 28 | 25 | | 26 | | 29 | 28 |
|  | 24 | 29 | 24 | | 27 | | 29 | 28 |
|  | 25 | 27 | 27 | | 26 | | 30 | 27 |
|  | 24.625 | 27.875 | 25.5 | | 26.125 | | 29.25 | 27.875 |
| Growth Rate | | | | NC | | NASH | | Treatment |
| 7 week vs 10 week | | | | 6.09%*** | | 4.93%** | | 9.31%*** |

Note: **（*p*<0.01）, ***（*p*<0.001）

Supplementary Table 5. Analysis of changes in body weight growth rate

of mice at 6W

|  | 9w | | | | 12w | | | |
| --- | --- | --- | --- | --- | --- | --- | --- | --- |
|  | NC | NASH | Treatment | | NC | | NASH | Treatment |
| Average weight | 25 | 27 | 27 | | 28 | | 31 | 29 |
|  | 26 | 31 | 26 | | 30 | | 32 | 27 |
|  | 26 | 28 | 26 | | 30 | | 28 | 29 |
|  | 23 | 26 | 27 | | 29 | | 29 | 27 |
|  | 26 | 29 | 27 | | 25 | | 28 | 27 |
|  | 27 | 26 | 27 | | 23 | | 30 | 28 |
|  | 26 | 27 | 28 | | 24 | | 32 | 28 |
|  | 27 | 30 | 26 | | 25 | | 29 | 29 |
|  | 25.750 | 28.000 | 26.750 | | 26.150 | | 29.875 | 28.000 |
| Growth Rate | | | | NC | | NASH | | Treatment |
| 7 week vs 10 week | | | | 3.88%*** | | 6.70%*** | | 4.67%*** |

Note: ***（*p*<0.001）

Supplementary Table 6. Analysis of Changes in Growth Rate of Liver Wet Weight

in Mice

|  | 6w | | | | 8w | | | |
| --- | --- | --- | --- | --- | --- | --- | --- | --- |
|  | NC | NASH | Treatment | | NC | | NASH | Treatment |
| Average weight | 1.52 | 1.88 | 1.77 | | 1.59 | | 2.03 | 1.82 |
|  | 1.53 | 1.84 | 1.71 | | 1.62 | | 1.99 | 1.88 |
|  | 1.51 | 1.85 | 1.72 | | 1.58 | | 1.92 | 1.85 |
|  | 1.58 | 1.77 | 1.69 | | 1.66 | | 2.06 | 1.87 |
|  | 1.49 | 1.81 | 1.73 | | 1.63 | | 1.98 | 1.84 |
|  | 1.46 | 1.72 | 1.65 | | 1.67 | | 1.95 | 1.91 |
|  | 1.59 | 1.88 | 1.68 | | 1.69 | | 1.97 | 1.79 |
|  | 1.47 | 1.83 | 1.75 | | 1.61 | | 2.03 | 1.86 |
|  | 1.51875 | 1.8225 | 1.7125 | | 1.63125 | | 1.99125 | 1.8525 |
| Growth Rate | | | | NC | | NASH | | Treatment |
| 6week vs 8 week | | | | 7.41%*** | | 9.26%*** | | 8.18%*** |

Note: ***（*p*<0.001）


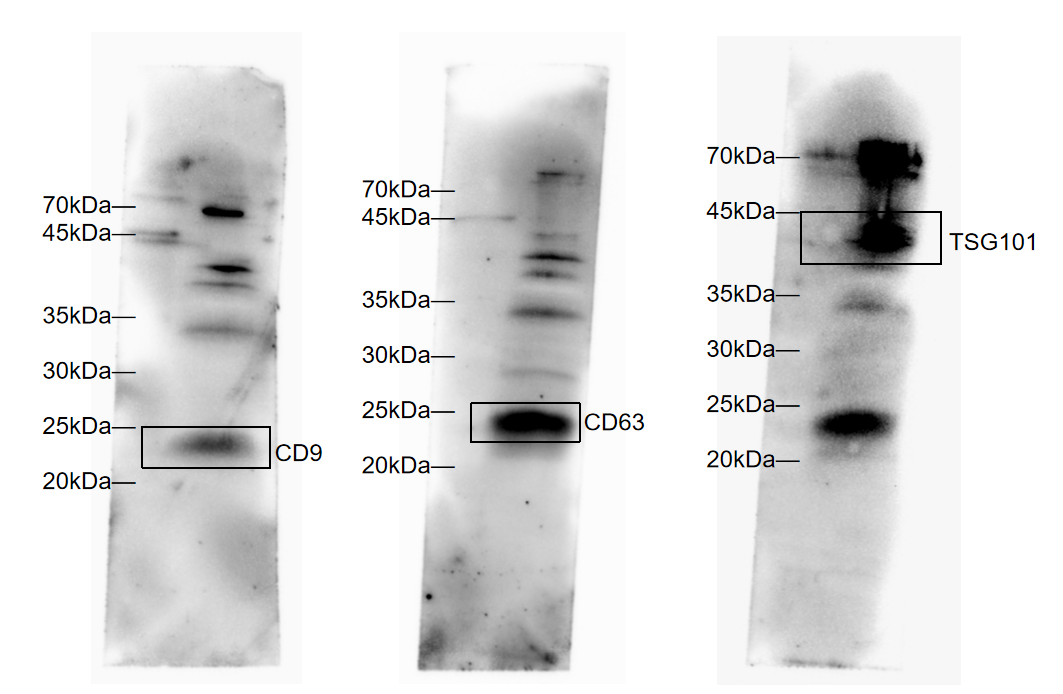
Supplementary Figure 27. Original full-length immunoblots of Expression of exosomes CD9, CD63 and TSG101 shown in Figure 3D. Immunoblots were exposed for 120 sec. The black wireframe represents Figure 3D


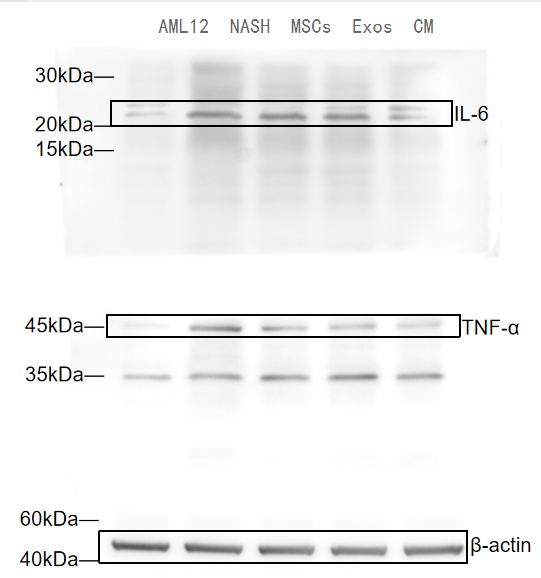


Supplementary Figure 28. Original full-length immunoblot for cellular levels of IL-6, TNF-α, and β-actin is shown in Figure 5. ( Note:The immunoblot was overexposed and exposed for 150 sec. The multiple exposure images can be seen in Supplementary Figure 37. The black wireframe represents Figure 5)


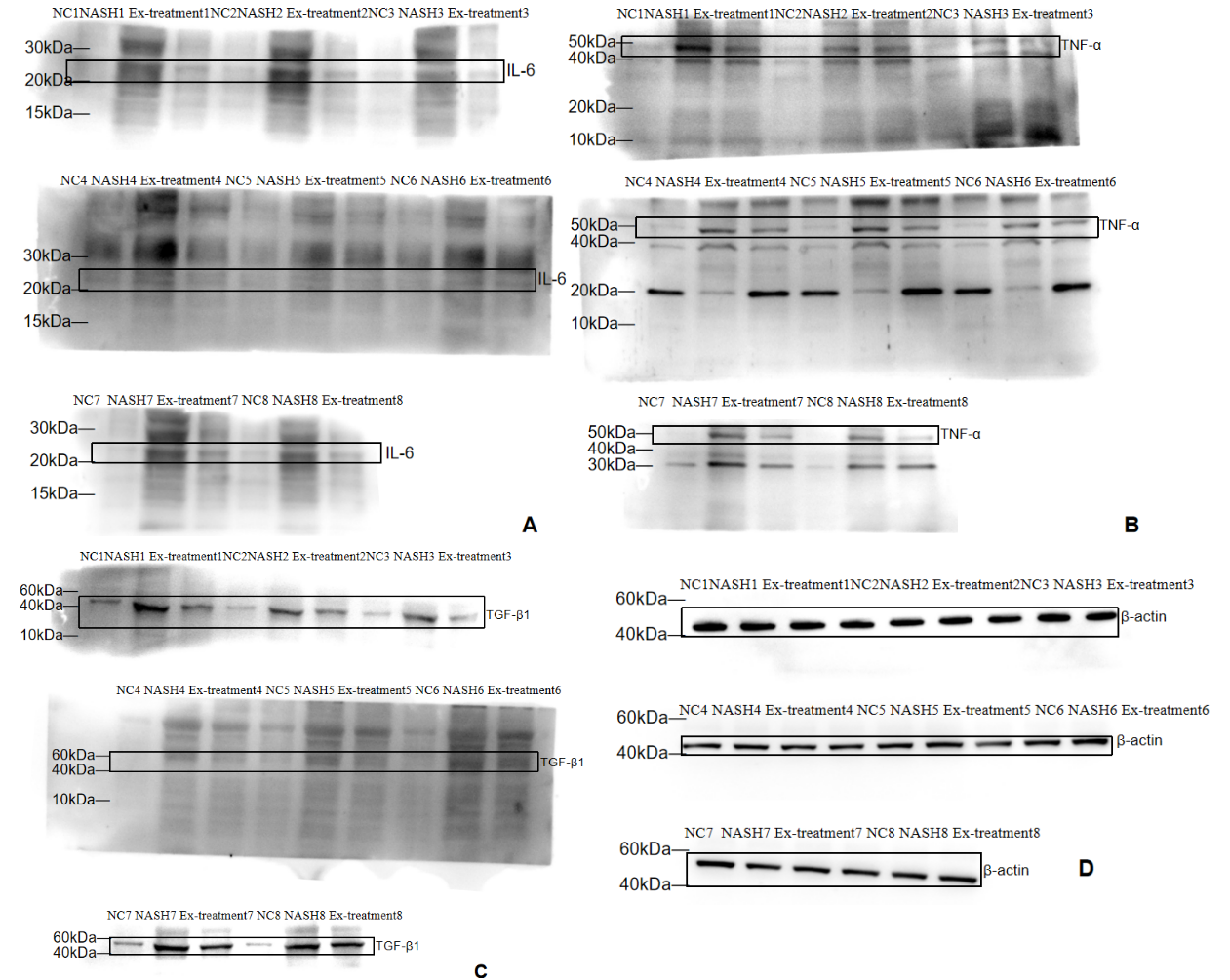


Supplementary Figure 29. Original full-length immunoblot for IL-6, TNF-α, TGF-β1and β-actin at animal level 6w are shown in Supplementary Figure 6. (Note:The immunoblot was overexposed and exposed for 120 sec. The multiple exposures of β-actin D group can be seen in Supplementary Figure 38. The black wireframe represents Supplementary Figure 6)


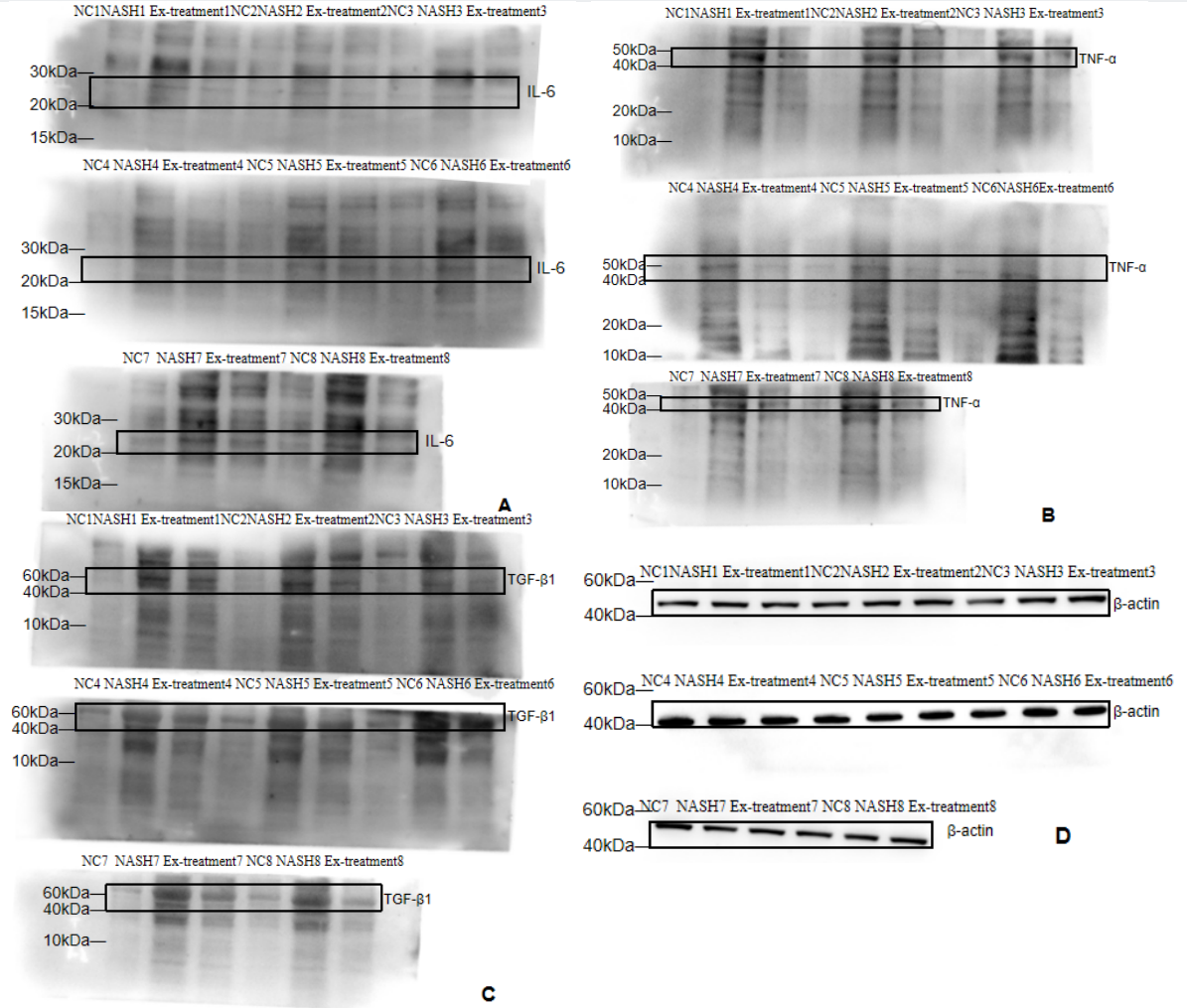


Supplementary Figure 30. Original full-length immunoblot for IL-6, TNF-α, TGF-β1and β-actin at animal level 8w are shown in Supplementary Figure 7. ( Note:The immunoblot was overexposed and exposed for 120 sec. An image of one of the multiple exposures of β-actin can be seen in Supplementary Figure 39. The black wireframe represents Supplementary Figure 7)


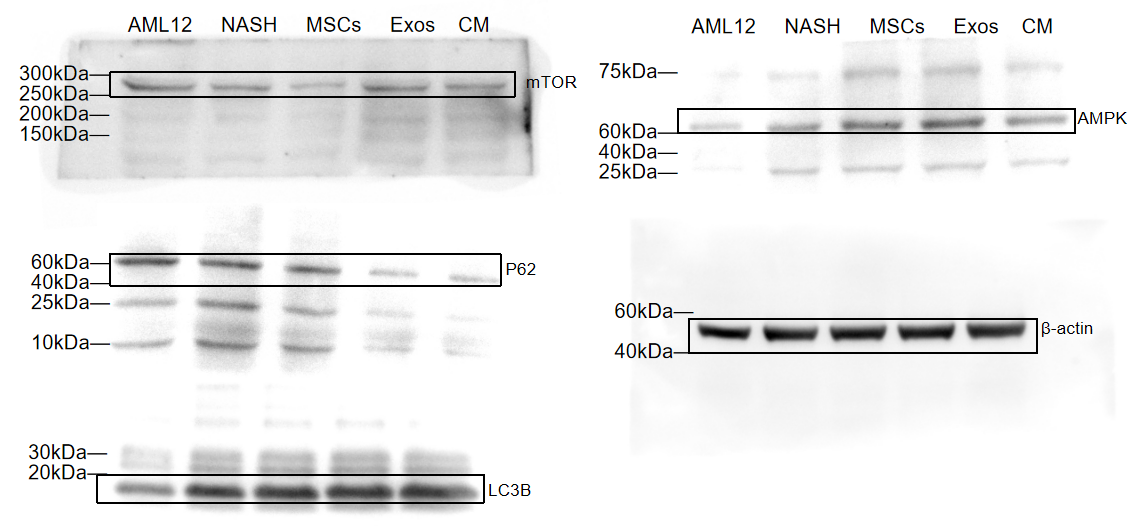
Supplementary Figure 31. Original full-length immunoblot for for cellular levels of mTOR, P62, LC3B, AMPK and β-actin is shown in Supplementary Figure 11. ( Note:The immunoblot was overexposed and exposed for 120 sec. An image of one of the multiple exposures of β-actin can be seen in Supplementary Figure 40. The black wireframe represents Supplementary Figure 11)


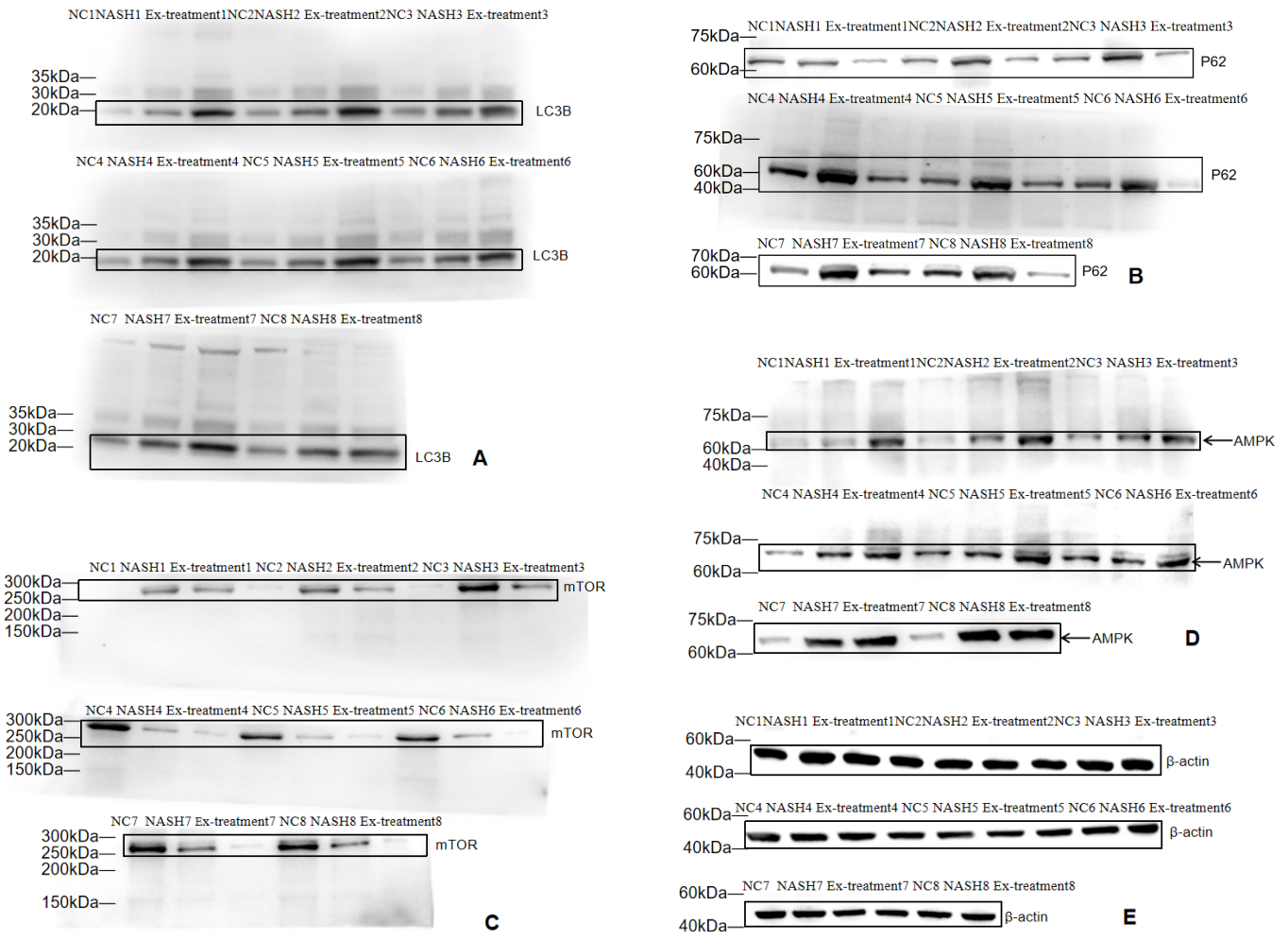


Supplementary Figure 32. Original full-length immunoblot for for LC3B, P62, mTOR, AMPK and β-actin at animal level 6w are shown in Supplementary Figure 16. ( Note:The immunoblot was overexposed and exposed for 120 sec. An image of one of the multiple exposures of β-actin can be seen in Supplementary Figure 41. The black wireframe represents Supplementary Figure 16)


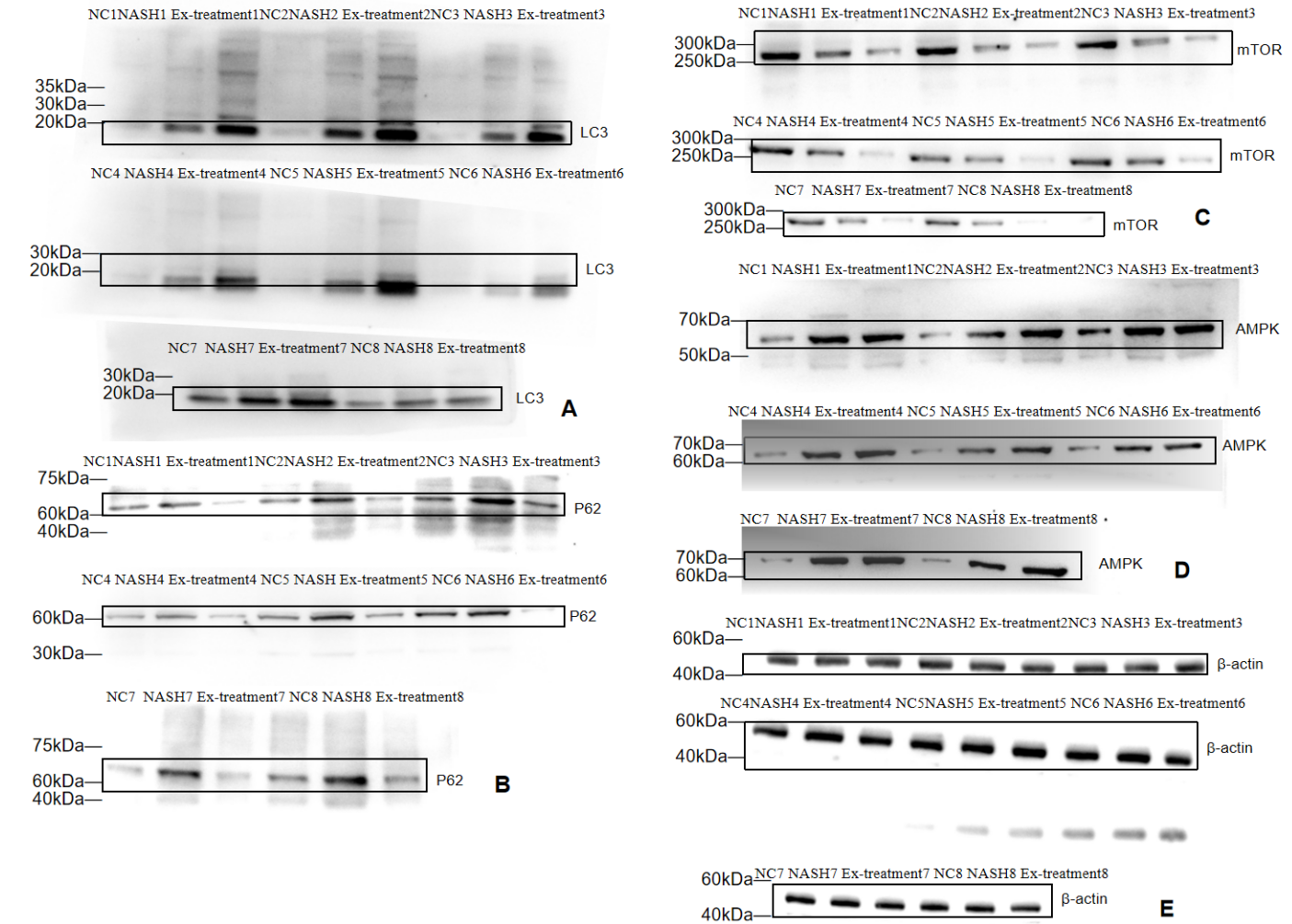


Supplementary Figure 33. Original full-length immunoblot for for LC3B, P62, mTOR, AMPK and β-actin at animal level 8w are shown in Supplementary Figure 17. ( Note:The immunoblot was overexposed and exposed for 120 sec. An image of one of the multiple exposures of β-actin can be seen in Supplementary Figure 42. The black wireframe represents Supplementary Figure 17)


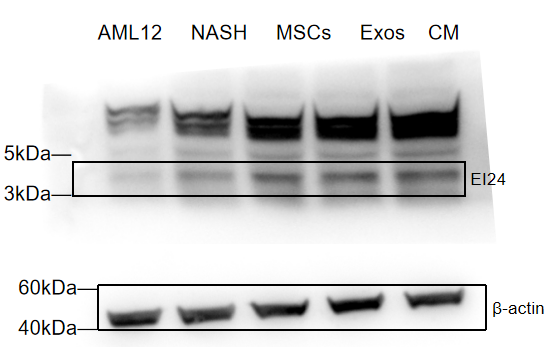


Supplementary Figure 34. Original full-length immunoblot for for cellular levels of EI24 is shown in Supplementary Figure 20. ( Note:The immunoblot was overexposed and exposed for 120 sec. An image of one of the multiple exposures of β-actin can be seen in Supplementary Figure 43. The black wireframe represents Supplementary Figure 20)


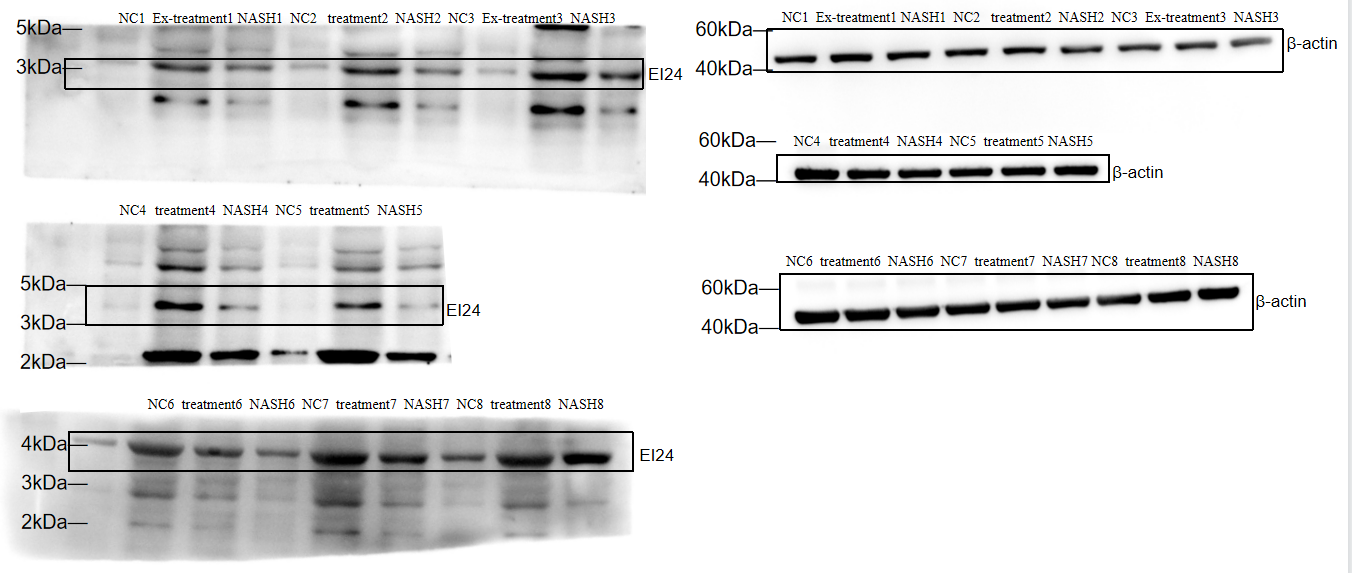


Supplementary Figure 35. Original full-length blot for for EI24 at animallevel 6w are shown in Supplementary Figure 24. ( Note:The immunoblot was overexposed and exposed for 120 sec. An image of one of the multiple exposures of β-actin can be seen in Supplementary Figure 44. The black wireframe represents Supplementary Figure 24)


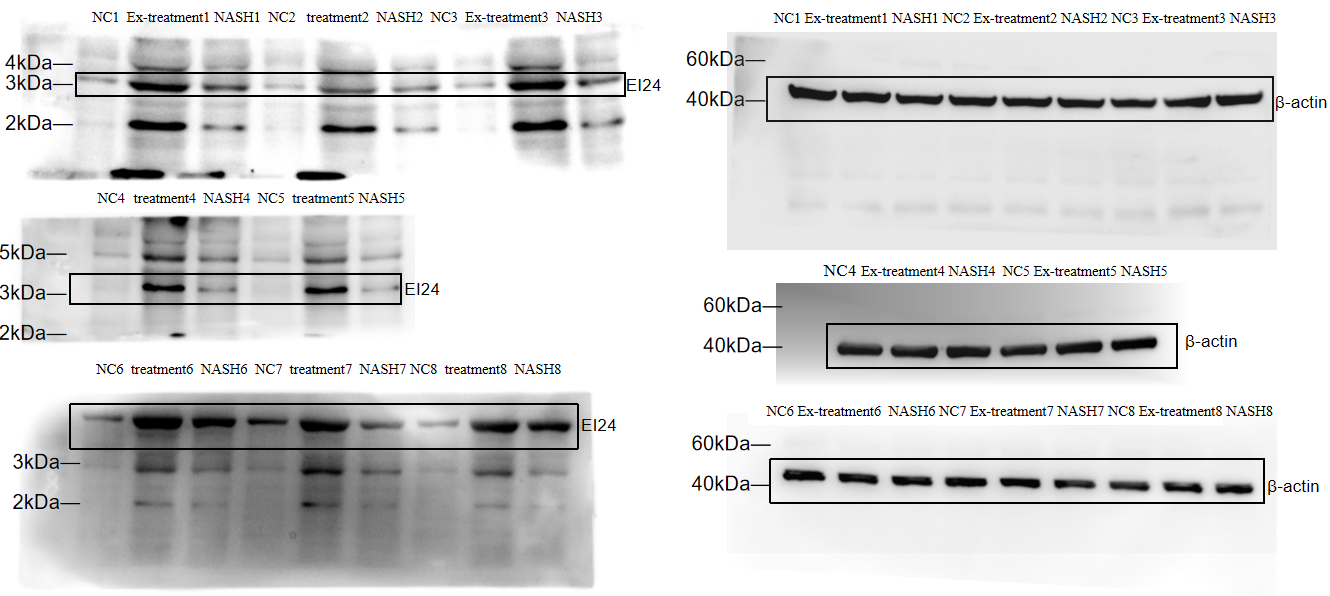
Supplementary Figure 36. Original full-length blot for for EI24 at animal level 8w are shown in Supplementary Figure 25. ( Note:The immunoblot was overexposed and exposed for 120 sec. The multiple exposures of β-actin can be seen in Supplementary Figure 45. The black wireframe represents Supplementary Figure 25)


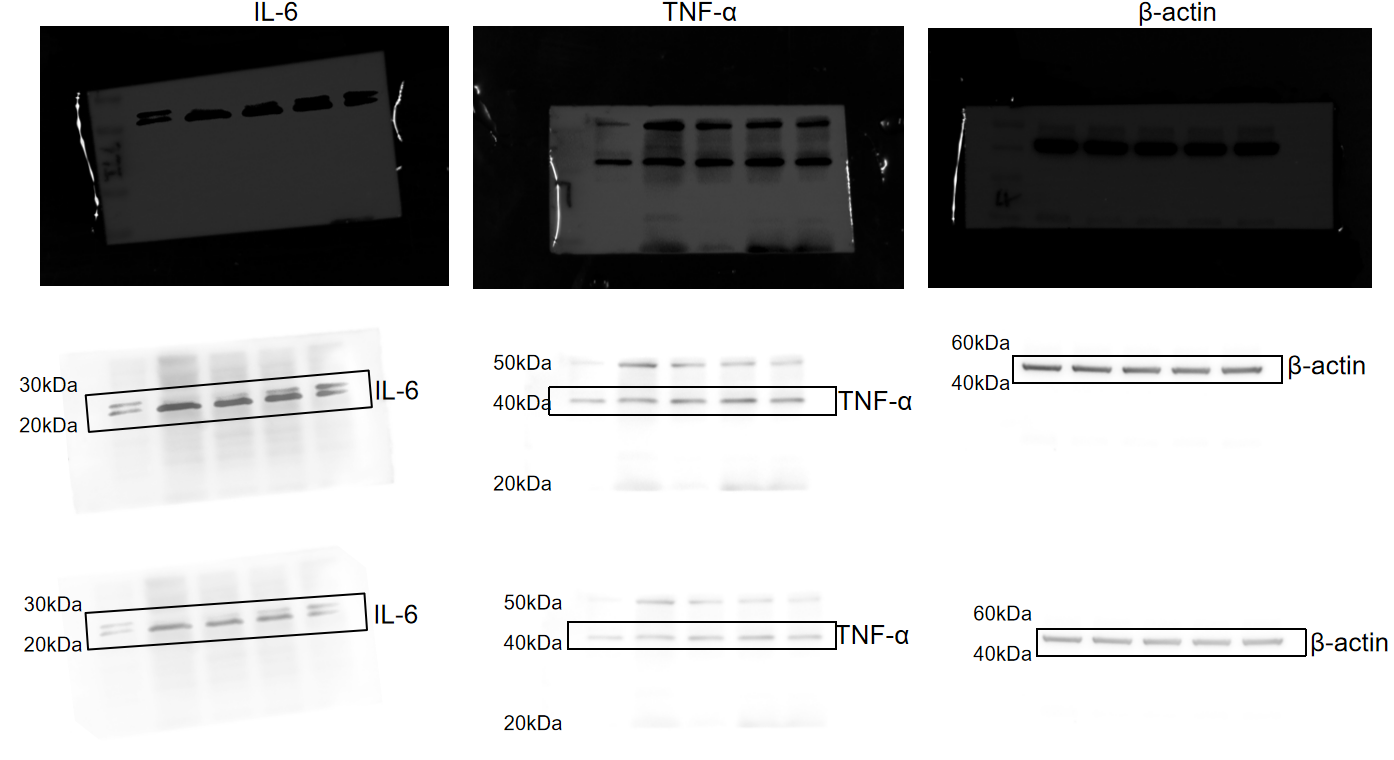


Supplementary Figure 37. The Multiple exposure images of the original full-length immunoblot of the cellular levels of IL-6, TNF-α, and β-actin; The sequence is 0 exposure, 150sec, 180sec


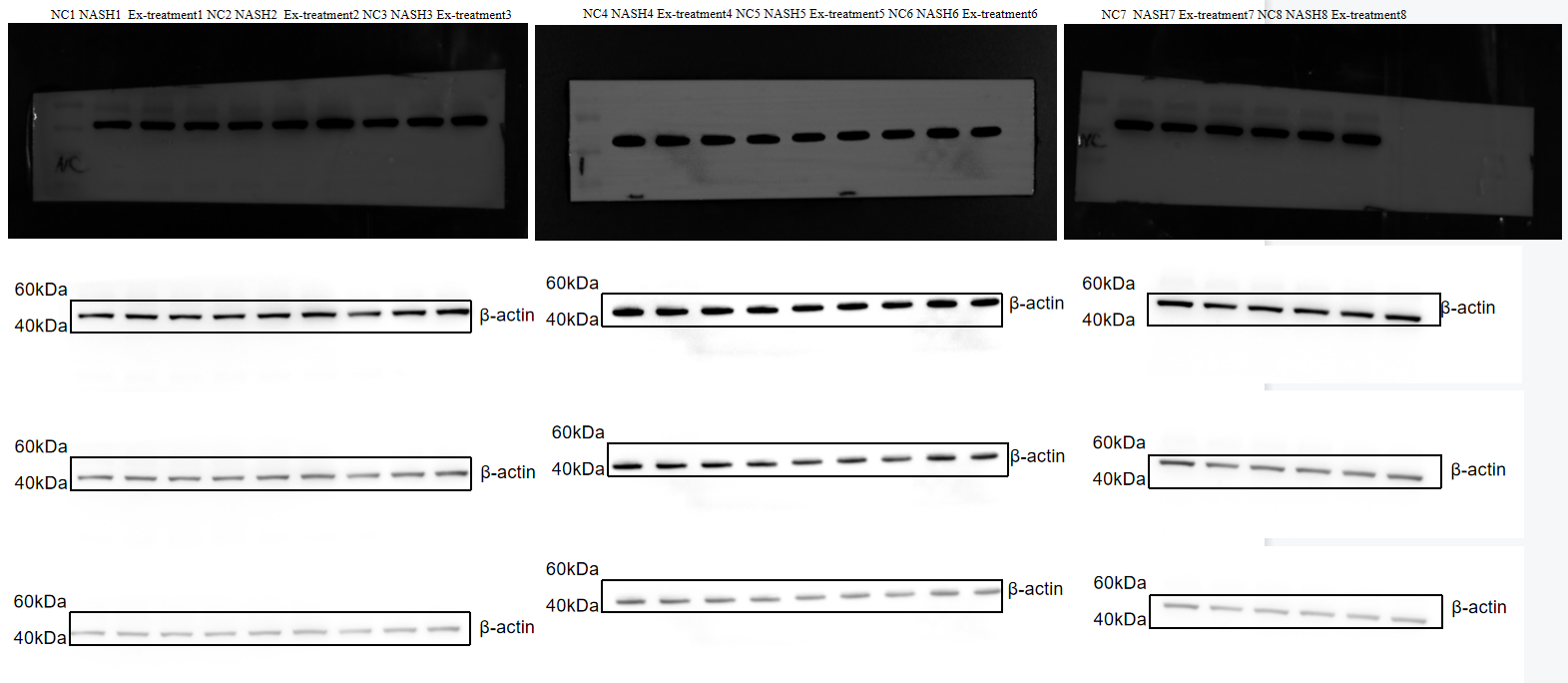


Supplementary Figure 38. The Multiple exposure images of the original full-length immunoblot of the 6W animal levels of β-actin; The sequence is 0 exposure, 150sec, 180sec


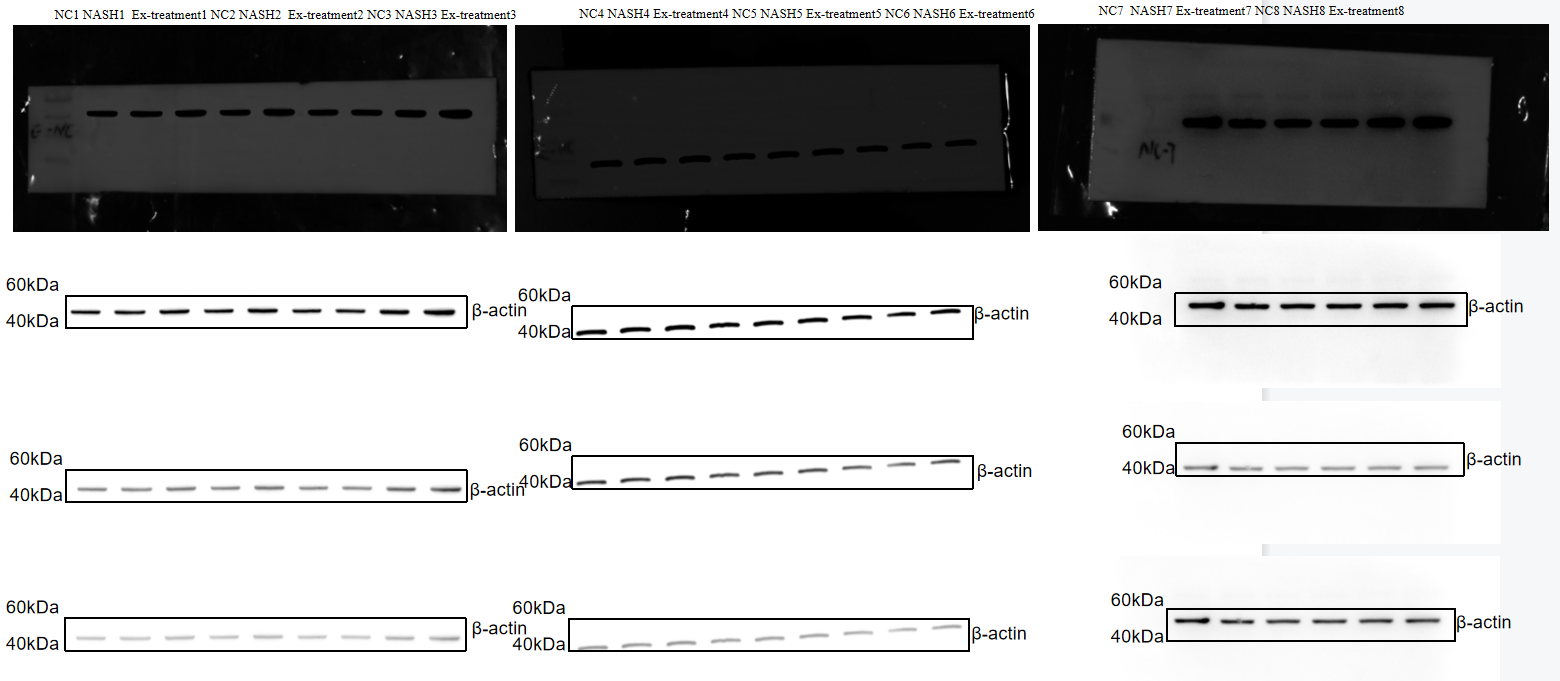


Supplementary Figure 39. The Multiple exposure images of the original full-length immunoblot of the 8W animal levels of β-actin; The sequence is 0 exposure, 150sec, 180sec


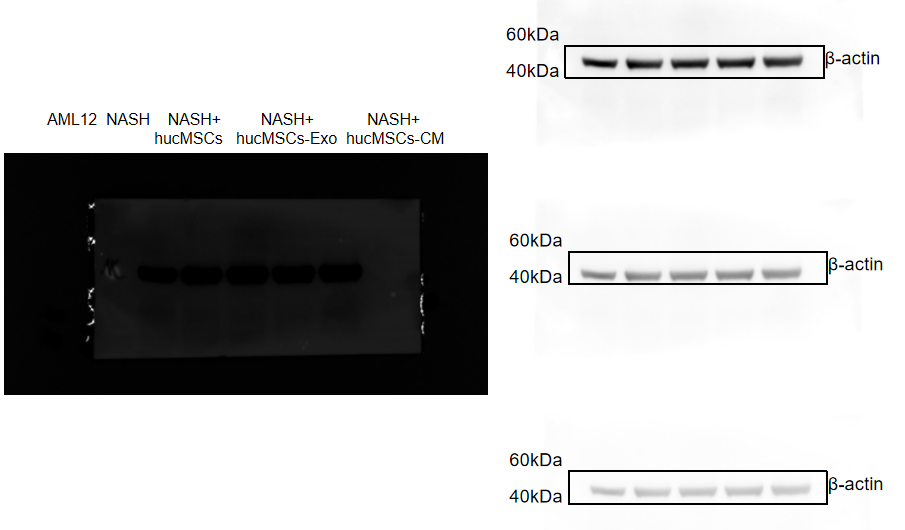
Supplementary Figure 40. The Multiple exposure images of the original full-length immunoblot of the cellular levels of β-actin; The sequence is 0 exposure, 150sec, 180sec


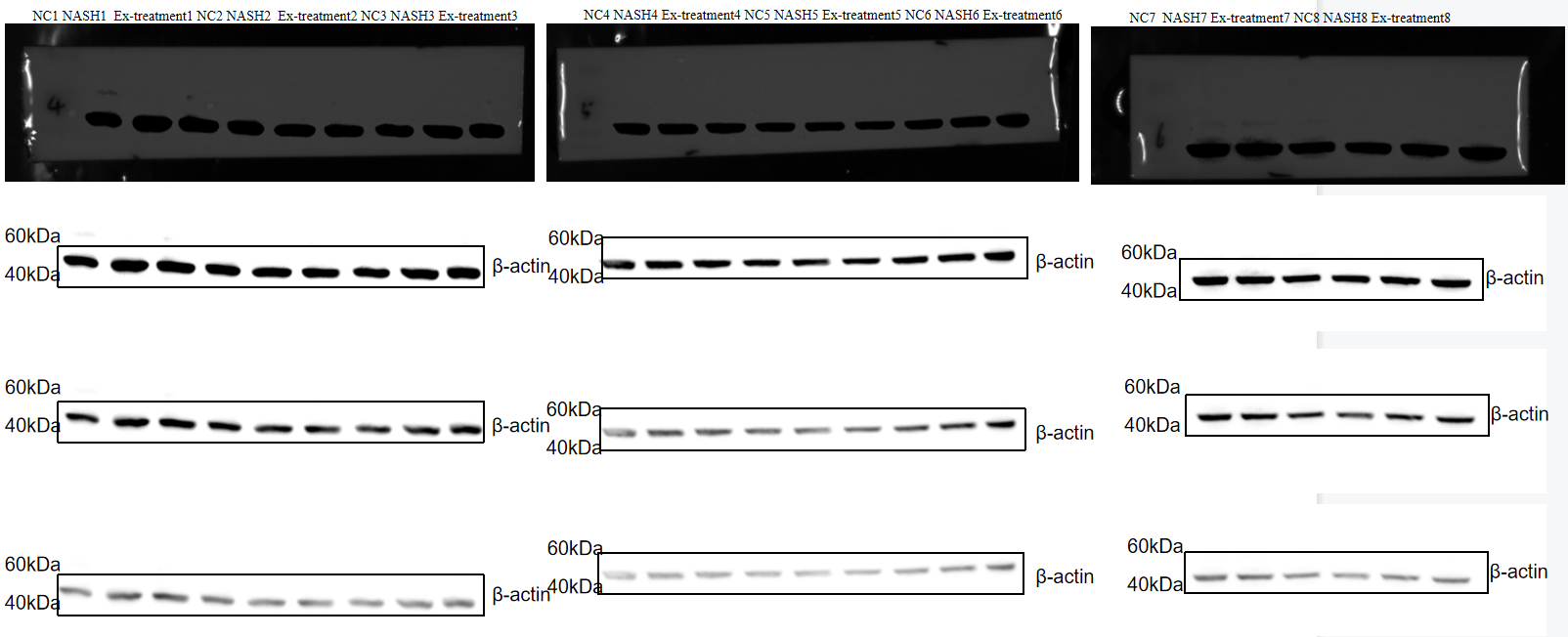
Supplementary Figure 41. The Multiple exposure images of the original full-length immunoblot of the 6W animal levels of β-actin; The sequence is 0 exposure, 150sec, 180sec


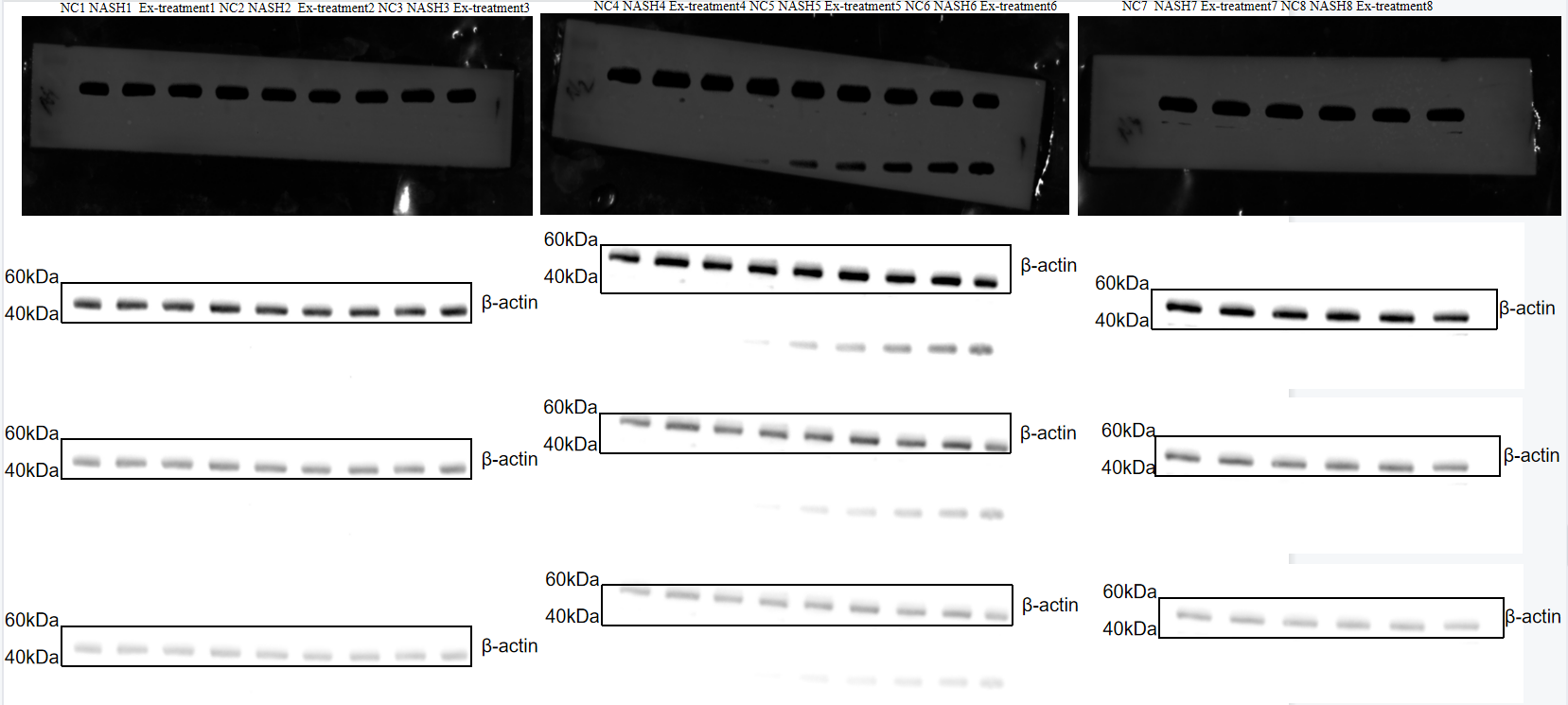
Supplementary Figure 42. The Multiple exposure images of the original full-length immunoblot of the 8W animal levels of β-actin; The sequence is 0 exposure, 150sec, 180sec


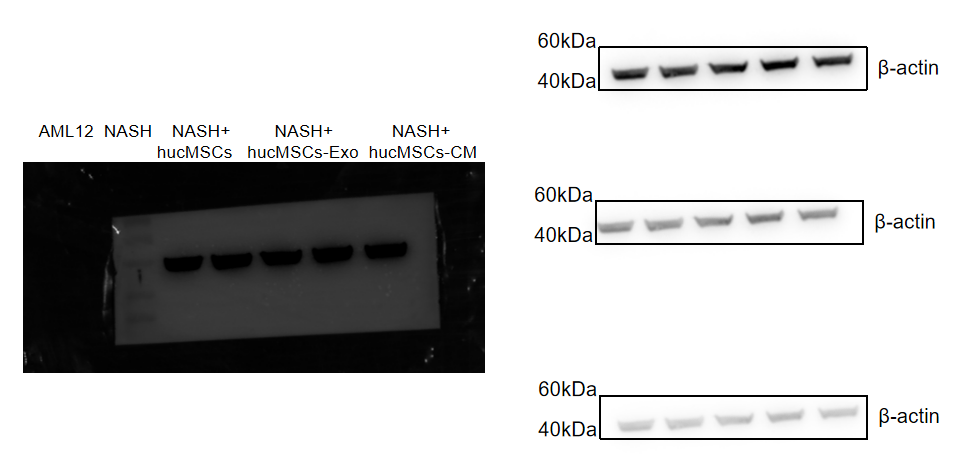


Supplementary Figure 43. The Multiple exposure images of the original full-length immunoblot of the cellular levels of β-actin; The sequence is 0 exposure, 150sec, 180sec


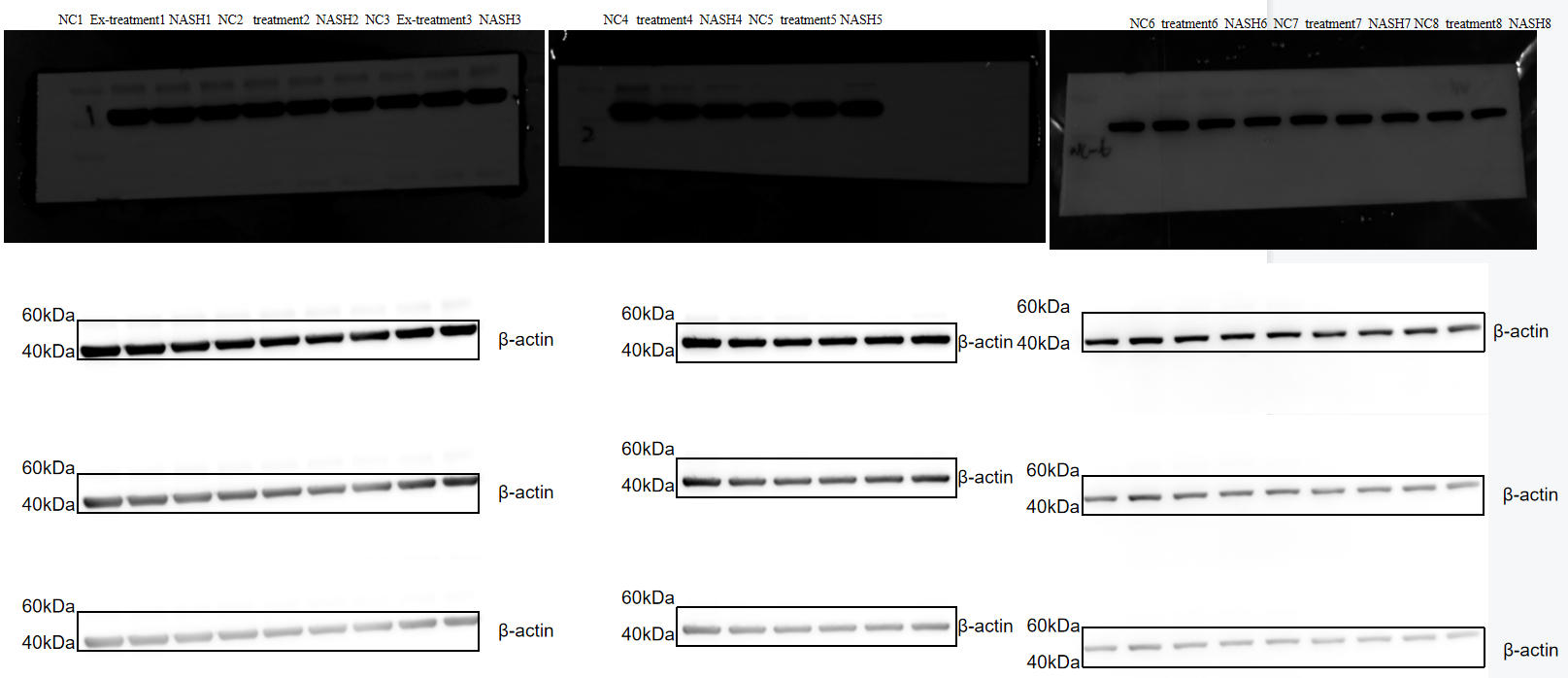
Supplementary Figure 44. The Multiple exposure images of the original full-length immunoblot of the 6W animal levels of β-actin; The sequence is 0 exposure, 150sec, 180sec


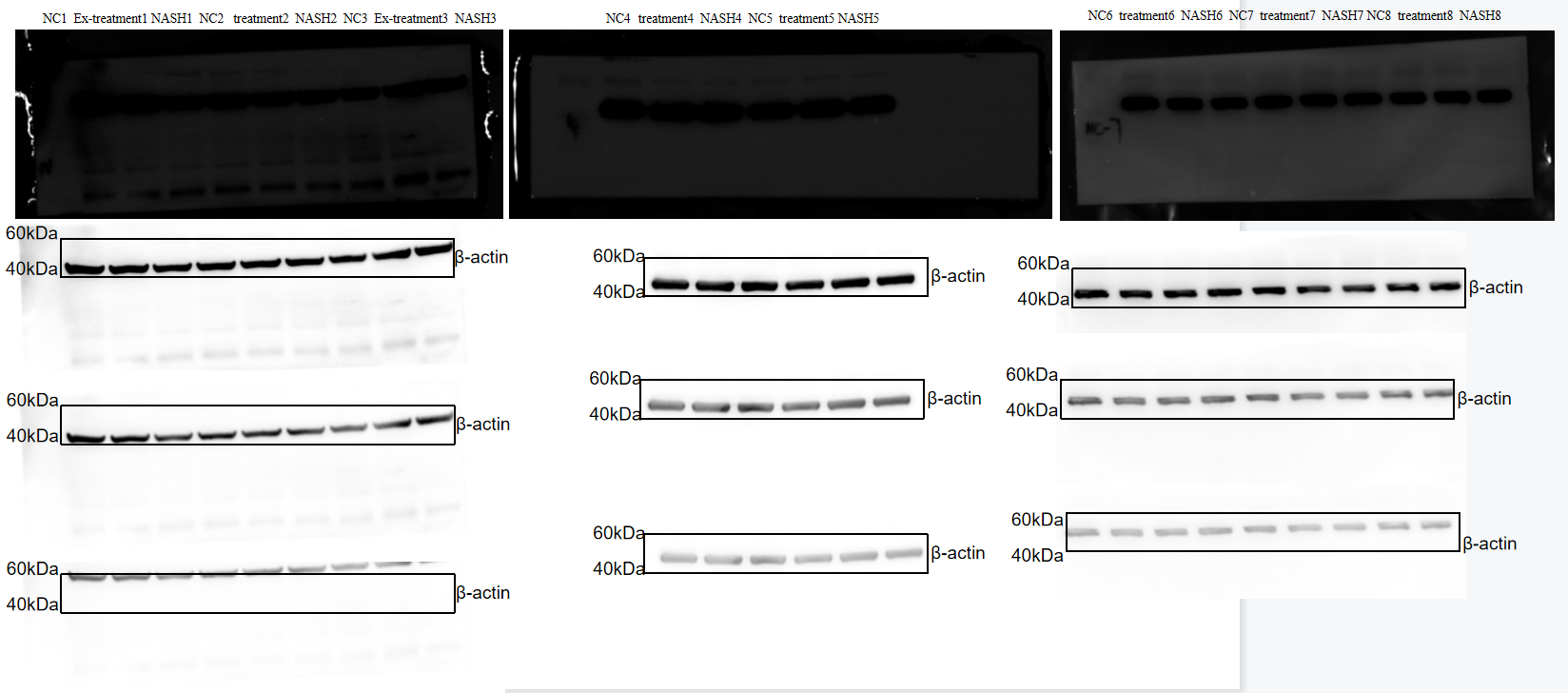


Supplementary Figure 45. The Multiple exposure images of the original full-length immunoblot of the 8W animal levels of β-actin; The sequence is 0 exposure, 150sec, 180sec
